# NCAPH drives breast cancer progression and identifies a gene signature that predicts luminal a tumour recurrence

**DOI:** 10.1002/ctm2.1554

**Published:** 2024-02-12

**Authors:** Marina Mendiburu‐Eliçabe, Natalia García‐Sancha, Roberto Corchado‐Cobos, Angélica Martínez‐López, Hang Chang, Jian Hua Mao, Adrián Blanco‐Gómez, Ana García‐Casas, Andrés Castellanos‐Martín, Nélida Salvador, Alejandro Jiménez‐Navas, Manuel Jesús Pérez‐Baena, Manuel Adolfo Sánchez‐Martín, María Del Mar Abad‐Hernández, Sofía Del Carmen, Juncal Claros‐Ampuero, Juan Jesús Cruz‐Hernández, César Augusto Rodríguez‐Sánchez, María Begoña García‐Cenador, Francisco Javier García‐Criado, Rodrigo Santamaría Vicente, Sonia Castillo‐Lluva, Jesús Pérez‐Losada

**Affiliations:** ^1^ Instituto de Biología Molecular y Celular del Cáncer (IBMCC‐CIC) Universidad de Salamanca/CSIC Salamanca Spain; ^2^ Biosanitary Research Institute of Salamanca (IBSAL) Salamanca Spain; ^3^ Departamento de Bioquímica y Biología Molecular Facultad de Ciencias Químicas Universidad Complutense Madrid Spain; ^4^ San Carlos Health Research Institute (IdISSC) Madrid Spain; ^5^ Biological Systems and Engineering Division Lawrence Berkeley National Laboratory (LBNL) Berkeley California USA; ^6^ Berkeley Biomedical Data Science Center Lawrence Berkeley National Laboratory (LBNL) Berkeley California USA; ^7^ Departamento de Medicina Universidad de Salamanca Salamanca Spain; ^8^ Servicio de Transgénesis Plataforma Nucleus Universidad de Salamanca Salamanca Spain; ^9^ Departamento de Anatomía Patológica Universidad de Salamanca Salamanca Spain; ^10^ Servicio de Anatomía Patológica Hospital Universitario de Salamanca Salamanca Spain; ^11^ Servicio de Oncología Hospital Universitario de Salamanca Salamanca Spain; ^12^ Departamento de Cirugía Universidad de Salamanca Salamanca Spain; ^13^ Departamento de Informática y Automática Universidad de Salamanca Salamanca España

**Keywords:** breast cancer, genetic signature, LASSO, luminal A subtype, NCAPH, prognosis, relapse‐free survival

## Abstract

**Background:**

Luminal A tumours generally have a favourable prognosis but possess the highest 10‐year recurrence risk among breast cancers. Additionally, a quarter of the recurrence cases occur within 5 years post‐diagnosis. Identifying such patients is crucial as long‐term relapsers could benefit from extended hormone therapy, while early relapsers might require more aggressive treatment.

**Methods:**

We conducted a study to explore non‐structural chromosome maintenance condensin I complex subunit H’s (NCAPH) role in luminal A breast cancer pathogenesis, both in vitro and in vivo, aiming to identify an intratumoural gene expression signature, with a focus on elevated NCAPH levels, as a potential marker for unfavourable progression. Our analysis included transgenic mouse models overexpressing NCAPH and a genetically diverse mouse cohort generated by backcrossing. A least absolute shrinkage and selection operator (LASSO) multivariate regression analysis was performed on transcripts associated with elevated intratumoural NCAPH levels.

**Results:**

We found that NCAPH contributes to adverse luminal A breast cancer progression. The intratumoural gene expression signature associated with elevated NCAPH levels emerged as a potential risk identifier. Transgenic mice overexpressing NCAPH developed breast tumours with extended latency, and in Mouse Mammary Tumor Virus (MMTV)‐*NCAPH^ErbB2^* double‐transgenic mice, luminal tumours showed increased aggressiveness. High intratumoural Ncaph levels correlated with worse breast cancer outcome and subpar chemotherapy response. A 10‐gene risk score, termed Gene Signature for Luminal A 10 (GSLA10), was derived from the LASSO analysis, correlating with adverse luminal A breast cancer progression.

**Conclusions:**

The GSLA10 signature outperformed the Oncotype DX signature in discerning tumours with unfavourable outcomes, previously categorised as luminal A by Prediction Analysis of Microarray 50 (PAM50) across three independent human cohorts. This new signature holds promise for identifying luminal A tumour patients with adverse prognosis, aiding in the development of personalised treatment strategies to significantly improve patient outcomes.

## Graphical abstract

- NCAPH significantly influences luminal A breast cancer progression and affects tumour behaviour and treatment response.
- GSLA10, a gene signature associated with NCAPH, shows promise in predicting luminal A tumour recurrence, demonstrating efficacy alongside existing models such as Oncotype DX.
- The study highlights the potential for tailored treatment approaches in luminal A breast cancer based on NCAPH and GSLA10 insights.

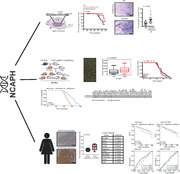

## 1 BACKGROUND

Breast cancer is currently the most frequently diagnosed tumour worldwide and the leading cause of cancer‐related deaths in women.^1^ In recent years, assays have been designed to evaluate the risk of breast cancer relapse based on the expression of multiple genes, thereby enabling the selection of the best treatment option.^2^, ^3^, ^4^, ^5^, ^6^, ^7^, ^8^ Through these tests, intrinsic subtypes of breast cancer can be defined to enhance the ability to predict the outcome of this disease.^5^, ^9^, ^10^, ^11^

One of the limitations of these signatures is that their long‐term predictive ability has not yet been fully determined, particularly in luminal A tumours.^10^, ^12^ Luminal A tumours have the most favourable prognosis, with the highest disease‐free survival (DFS) rate at 10 years; however, it should be noted that 25% of these tumours that relapse do it within the first 5 years, and the relapse risk notably triples between the 5th and 10th years.^13^ Furthermore, luminal A tumours are most prone to recurrence beyond the 10‐year follow‐up mark.^14^ Indeed, metastasis may appear many years after the diagnosis of luminal A breast cancer and even decades after the removal of the primary tumour.^15^, ^16^ Additionally, treated luminal A tumours that eventually relapse may evolve into aggressive metastatic cancers.^17^ Therefore, it is paramount to identify luminal A tumours with a heightened relapse risk in the short to medium term, as these may require a more aggressive therapeutic approach. Similarly, it is essential to identify patients at an elevated risk of long‐term recurrence to enable meticulous monitoring and potentially extend their secondary chemoprevention hormone therapy.^14^ Thus, there is a clear need to identify gene signatures for tumours already defined as luminal A that could distinguish those with potentially worse outcome in short‐, medium‐ and long‐term follow‐up. Indeed, including genes that are involved in the pathogenesis of luminal A tumours with poor outcome is likely to be useful for obtaining signatures that may more accurately predict prognosis.^18^ In this sense, luminal A tumours with a worse prognosis would exhibit higher genomic instability, including multiple modifications to the focal copy number^19^ and alterations in genes involved in mitosis.^20^

*NCAPH* encodes a member of the Barr protein family that contributes to the condensin complex. Condensins are large protein complexes that assemble interphase chromatin into chromosomes and organise their segregation during mitosis and meiosis.^21^ Two condensin complexes have been described,^22^ of which the condensin II complex is predominantly located in the nucleus during interphase and binds to chromosomes during mitosis.^23^, ^24^, ^25^ Conversely, the condensin I complex is located in the cytoplasm and binds to chromosomes after rupture of the nuclear membrane.^26^ However, a small fraction of condensin I has been detected in the nucleus during interphase, where it helps regulate gene expression and chromosome condensation.^26^ These two multiprotein complexes share two central subunits: the structural chromosome maintenance proteins SMC2 and SMC4. They also contain three non‐structural chromosome maintenance (SMC) subunits: NCAPD2, NCAPG and non‐SMC condensin I complex subunit H (NCAPH) in the condensin I complex and NCAPD3, NCAPG2 and NCAPH2 in the condensin II complex. The NCAPH and NCAPH2 subunits belong to the kleisin protein family,^27^, ^28^, ^29^ and mutations in the latter have been implicated in genomic instability.^30^

Here, we demonstrate that NCAPH participates in the pathogenesis of breast cancer and in chemotherapy resistance in vivo and in vitro. MMTV‐*NCAPH^ErbB2^* double‐transgenic mice overexpressing NCAPH generated more aggressive breast tumours, and in a cohort of genetically heterogeneous transgenic mice generated by backcrossing, these tumours had a worse outcome and a poor response to chemotherapy. Moreover, using the least absolute shrinkage and selection operator (LASSO) multivariate regression model,^31^ we identified a group of 10 genes associated with high intratumoural NCPAH levels from this cohort of mice that formed a signature capable of defining patients with a worse outcome of luminal A tumours, better defining those who could benefit from more personalised treatment.

## 2 METHODS

### 2.1 Patient samples and immunostaining

Human primary breast tumours were collected at the University Hospital of Salamanca, after the Hospital's Institutional Ethics Review Board approved the protocols for the collection and use of patient samples. Written informed consent was obtained from all patients to conduct the study on these tumour samples. NCAPH expression was evaluated in a retrospective study of 28 human tumour samples by immunohistochemistry (IHC), eight of which were from patients who had a poor outcome and developed liver metastases, while the other 20 had a good outcome. IHC was performed automatically using the Bond Polymer Refine Detection kit (BOND III: Leica, Biosystems, Leica Microsystems), probing the sections for 60 min with an antibody against NCAPH (HPA0030008, Sigma–Aldrich) diluted 1:100 and then counterstained with haematoxylin. Appropriate positive and negative controls were used.

Fiji Is Just ImageJ (FIJI) software was used to analyse NCAPH expression in human breast tumours.^32^ FIJI, is an open‐source, Java‐based image processing platform. Tailored for biological image analysis, FIJI provides a comprehensive suite of tools for advanced processing and quantitative assessment, including densitometry. This software ensures precise and replicable analysis of microscopic images,^32^ sampling seven fields for each tumour randomly with a Leica ICC50 HD camera at 40× magnification under the control of the Leica Application Suite V3.7 software. The mean intensity (MI) of the epithelial cells was obtained and the reciprocal intensity (RI) was calculated in each field by subtracting the epithelial MI from the background.^33^ The normalised RI was obtained by dividing the RI by the background value, and the mean normalised RI of the seven fields was calculated for each patient. For more details on image analysis using FIJI software, see the Supporting Information.

### 2.2 NCAPH transgenic mice

All mice were housed at the Animal Research Facility of the University of Salamanca and all procedures were approved by the Institutional Animal Care and Bioethical Committee. The mice were maintained in ventilated filter cages under specific pathogen‐free conditions and fed ad libitum. MMTV‐*NCAPH* mice were generated at the Transgenic Facility of the University of Salamanca. A 2226 bp fragment containing the entire human *NCAPH* coding region was generated by PCR, cloned, and inserted into the *EcoRI* site of the pMKbpAII plasmid containing the Mouse Mammary Tumour Virus (MMTV) promoter. The construct containing *NCAPH* free of any vector sequence (BssHII fragment) was injected into fertilised oocytes extracted from FVB animals. Mice were screened for the presence of the transgene in the Southern blots of tail DNA digested with *XhoI*. The blots were hybridised with the same *XhoI* DNA fragment (658 bp) and confirmed by qPCR performed on mammary gland tissue of 3‐month‐old mice. Two founders (*NCAPH #1* and *NCAPH #2*) were obtained and bred, and their tail DNAs were genotyped using PCR. Three MMTV‐*NCAPH* cohorts were generated: one cohort of nulliparous mice (*N* = 10) and two cohorts of parous mice that were pregnant twice: MMTV‐*NCAPH* #1 (*N* = 29) and *NCAPH* #2 (*N* = 29). FVB/N‐Tg(MMTVneu)202Mul/J mice carrying the *avian erythroblastosis oncogene B2/neuroblastoma‐derived* (*ErbB2/cNeu)* protooncogene (*ErbB2* after that) under the control of the MMTV promoter (MMTV‐*Erbb2* transgene)^34^ were obtained from Jackson Laboratory. A new cohort of mice was obtained by crossing MMTV‐*NCAPH* and MMTV‐*ErbB2* mice to obtain dual transgenic mice (*N* = 33).

### 2.3 Backcrossing and F1 allografts

A genetically heterogeneous mouse cohort was generated by backcrossing two inbred strains as previously described.^35^ Briefly, we crossed the breast cancer‐resistant C57BL/6 mouse strain (C57) with an *FVB/N‐Tg(MMTVneu)202Mul/J* susceptible strain (FVB). F1‐*Neu^+^* males generated with the transgene were mated with FVB non‐transgenic females to obtain a backcrossed cohort of MMTV‐*ErbB2* mice (BX‐*Neu^+^*) (*N* = 147). The concentration of tail DNA was measured using a Nanodrop ND‐1000 Spectrophotometer and used for genotyping.^35^

Tumour cells from BX‐*Neu^+^* mice were transplanted into F1 female recipients and 100 μL of a single‐cell suspension containing 2−5 × 10^6^ cells was injected into both inguinal flanks of each mouse. Each tumour was transplanted into two individuals (*N* = 125). Chemotherapy was initiated when the tumour diameter reached 12 mm by treating 58 mice with docetaxel (25 mg/kg; Taxotere, Sanofi Aventis) and 69 mice with doxorubicin (5 mg/kg; Farmiblastina, Pfizer), and each drug was injected intraperitoneally (IP). Docetaxel and doxorubicin were administered every 8 and 10 days, respectively, and the mice were sacrificed when the tumour reached 25 mm in diameter or 2 months after the end of the treatment.

### 2.4 Histological analysis

Tumours and mammary glands were fixed in 4% paraformaldehyde (PFA: Scharlau FO) for 24 h at room temperature and washed in 70% ethanol before being embedded in paraffin for automated processing (Shandon Excelsior, Thermo). The samples were sectioned and stained with haematoxylin and eosin to evaluate their pathology under a microscope. Five photos (10× magnification) were taken randomly with a Leica ICC50 HD camera under the control of Leica Application Suite V3.7 software, quantifying the relative ductal area of the mammary glands. Image analysis was performed using *ImageJ*, selecting the ductal area (epithelial cells forming the duct) while excluding the adipose tissue and duct lumen. The ductal epithelial area was divided by the total field area to calculate the relative percentages, and the mitotic index of the tumours was defined as very high if there were more than eight mitoses at 40× magnification, high if there were between four and eight mitoses, moderate when there were between two and four mitoses, and low if there were less than two mitoses. A pathologist evaluated this parameter in the Pathology Unit of our Centre.

### 2.5 Immunostaining of mouse tissue

Immunostaining of mouse tissues was performed at the Pathology Unit of our Centre. Mammary gland or tumour sections (3 μm) were deparaffinised and probed with a primary antibody against Ki‐67 (MAD020310Q at a 1:50 dilution: Master Diagnostica) using Discovery ULTRA (Roche). The secondary antibody used was OmniMap anti‐Rb horseradish peroxidase (HRP) (#05269679001, Roche), and Ki‐67^+^ cells were quantified using Leica Application Suite V3.7 software with five selected areas on the slide at 20× magnification.

### 2.6 Cell culture

MCF‐7 and BT549 cells were grown in complete DMEM containing 4.5 g/L glucose and L‐glutamine (Sigma–Aldrich) at 37°C in a humid atmosphere containing 5% CO_2_. The medium was supplemented with 56 IU/mL penicillin, 56 mg/L streptomycin (Invitrogen), and 10% foetal bovine serum (LINUS #16sV30180.03), and all cell cultures were routinely tested for mycoplasma contamination. The cell lines were analysed for authentication at the Genomics Core Facility at the Instituto de Investigaciones Biomédicas ‘Alberto Sols’ (CSIC‐UAM) using STR PROFILE DATA, the STR amplification kit (GenePrint 10 System, Promega), STR profile analysis software GeneMapper v3.7 (Life Technologies), and a Genomic Analyser System ABI 3130 XL (Applied Biosystems). See the Supporting Information section for procedures related to cell viability, soft agar assays, Boyden chamber cell migration assays and qPCR assays.

### 2.7 Protein analysis

Proteins were analysed by Western blotting as previously described.^35^ In brief, proteins were extracted from the tumours in RIPA buffer (150 mM NaCl, 1% [v/v] NP40, 50 mM Tris–HCl [pH 8.0], .1% [v/v] SDS, 1 mM EDTA, .5% [w/v] deoxycholate), whereas the proteins from the cell lines were extracted in TNES buffer (100 mM NaCl, 1% [v/v] NP40, 50 mM Tris–HCl [pH 7.6], 20 mM EDTA), both buffers containing protease and phosphatase inhibitor cocktails (Sigma‒Aldrich; #P8340). The recovered proteins were quantified using a Bradford Protein Assay (Bio‐Rad, #5000006), resolved by SDS‐PAGE on 10 or 12% gels (Bio‐Rad, #456‐8085), and transferred to polyvinylidene difluoride membranes (Immobilon‐P, Millipore). The membranes were probed with the following primary antibodies raised against NCAPH (1:10000, #TA303239: OriGene), cyclin D1 (1:1000, #sc8396: Santa Cruz Biotechnology), γH2AX (1:200, Ser139 #05‐636: Millipore), pCHK1 (1:200, Ser345 #2348: Cell Signalling), tubulin (1:1000, DM1A; Sigma‒Aldrich; #T6199), HSP90 (1:1000, #515081: Santa Cruz Biotechnology), actin (1:1000, #A5441: Sigma‒Aldrich), pERK1/2 (1:1000, #9101: Cell Signalling), total ERK1/2 (1:1000, #4696: Cell Signalling), pAKT S473 (1:1000, #32581: Elabscience), total AKT (1:1000, #30471: Elabscience), cleaved Caspase‐3 (Cell Signalling #9661), anti‐ERBB2 (1:1000, #ab2428: Abcam) and anti‐phospho‐ERBB2 (Tyr1248) (1:1000, #ab47755: Abcam). Antibody binding was detected using HRP‐conjugated anti‐mouse or anti‐rabbit secondary antibodies (1:10,000 dilution; Amersham) and visualised by enhanced chemiluminescence (ECL, #170‐5061: Bio‐Rad). Images were acquired using an ImageQuant LAS 500 Chemiluminescence CCD camera (GE Healthcare Life Sciences).

### 2.8 Inducible system for NCAPH

An inducible mammalian expression construct encoding NCAPH was obtained by cloning *NCAPH* into a pRetroX‐Tight‐Puro vector (Clontech #632104). The MCF‐7 and BT‐549 inducible *NCAPH* systems were generated by co‐transfecting pRetroXTet‐On Advanced with pRetroX‐Tight‐Puro‐*NCAPH*. *NCAPH* expression was induced in cells after exposure to doxycycline (10 μg/mL).

### 2.9 Identification of differentially expressed genes and functional enrichment analysis

The quality and quantity of the total RNA isolated from the cells were determined using an Agilent 2100 Bioanalyser and NanoDrop ND‐1000. Affymetrix GeneChip mouse gene 1.0 ST arrays were used, according to the manufacturer's protocol. Gene expression data for mouse breast cancers are available from the Gene Expression Omnibus (accession number GSE54582).^35^ The differentially expressed genes (DEGs) between animals with *Ncaph* high and *Ncaph* low in the BX‐*Neu* ^+^ cohort were identified using Transcriptome Analysis Console software, using a cutoff change (|log FC| > 2.0) and adjusted *p* ≤ .05. Gene Ontology (GO) pathway enrichment analyses were performed using the R package ‘clusterProfiler’.^36^, ^37^

The DEGs obtained in BX‐*Neu* ^+^ mice were analysed by LASSO regression using the R package ‘glmnet’,^31^ and the lambda value was determined by cross‐validation to penalise collinearity among genes. The animals were divided into three groups (high, medium and low risk) and their scores were calculated using the following equation:

$$\mathrm{Risks}\;\mathrm{core}=\sum_{i=0}^{n}\mathrm{ex}p_{i}\times \beta_{i}$$

where *exp*_*i*_ indicates each genes's expression level. Kaplan‒Meier (KM) lifespan analysis was performed to compare the prognostic differences between the three groups (R package: ‘survival’^38^, ^39^ and ‘survminer’^40^). For human analysis, we studied a combined patient database using Gene Expression‐based Outcome for Breast Cancer Online (GOBO),^41^ an online tool that downloads gene expression levels from an 1881‐sample breast cancer dataset. We used the GSE1456, GSE2603, GSE6532, GSE3494, GSE4922, GSE6532, GSE7390, GSE11121, GSE12093, GSE2034 and GSE5327 databases to select luminal A samples (401 patients). We first conducted a univariate Cox regression analysis of DEGs and then selected candidate genes related to relapse‐free survival (RFS) according to a criterion of *p*‐value <.25.^42^, ^43^ In multivariate analysis, a lenient *p*‐value threshold (e.g., *p* ≤ .25) is often used for variable selection, as advocated by Dr. Frank E. Harrell Jr. in ‘Regression Modeling Strategies’.^43^ This approach ensures that potentially significant predictors are not excluded prematurely despite their weaker associations in bivariate analyses. This inclusive initial selection, followed by refinement through techniques such as stepwise regression, aims to create a model that is comprehensive yet parsimonious, effectively capturing data relationships. The selected genes obtained in the univariate analysis were further analysed by LASSO regression using glmnet, and the regression coefficient lambda was determined by cross‐validation. Specifically, the GOBO cohort was randomly split into a training set (70%) to develop the model and a test set (30%) for validation. We then identified 10 genes that helped define disease prognosis using the LASSO model, which we referred to as the Gene Signature for Luminal A 10 (GSLA10). For the KM analysis of RFS, the patients were divided into three groups (high, medium and low risk), and the score was calculated using the aforementioned equation to compare the prognostic difference between the three groups. The results were then verified using receiver operating characteristic (ROC) curve analysis.^44^ Finally, we validated our model using two independent databases, Molecular Taxonomy of Breast Cancer International Consortium (METABRIC, 718 luminal A breast cancer patients)^45^ and The Cancer Genome Atlas Breast Invasive Carcinoma (TCGA‐BRCA, 499 luminal A breast cancer patients),^46^ both of which are available from cBioPortal (https://www.cbioportal.org/). In comparison with the Oncotype DX signature, we also constructed an Oncotype DX model. According to Paik et al., the expression levels of each Oncotype gene were normalised against a set of five reference genes: *ACTB*, *GAPDH*, *GUS*, *RPLPO* and *TFRC*. This normalisation involved measuring each gene's expression and then adjusting it by subtracting the mean expression value of these five reference genes.^6^ In both the training cohort (GOBO) and independent validation cohorts (METABRIC and TCGA‐BRCA), we evaluated, validated and compared the ability of GSLA10 and Oncotype to define RFS (GOBO) and DFS (METABRIC and TCGA‐BRCA) in luminal A tumours. During independent validation, both the GSLA10 and Oncotype pre‐trained models from GOBO were deployed without any modifications.

The concordance index (*C*‐index) is a statistical tool used to assess the predictive accuracy of a prognostic model, particularly in survival analysis. It is calculated by forming all possible pairs of subjects, evaluating whether the predicted survival times align with the actual survival outcomes, and excluding pairs with indeterminate ordering due to censoring or ties. The *C*‐index is the ratio of concordant pairs to the total number of evaluable pairs. Values range from .5 (no predictive ability) to 1.0 (perfect prediction), with higher values indicating better model performance.^47^, ^48^

### 2.10 Statistical analysis

Depending on the data distribution, we calculated either the Pearson or Spearman correlation coefficients or performed a Student's *t*‐test or Mann–Whitney *U*‐test to compare continuous variables between the two groups. ANOVA, or the Kruskal–Wallis test, was used to compare continuous variables across more than two groups. We used the KM estimator and log‐rank test to compare temporal variables. For contingency analysis, we used Fisher's exact test to analyse 2 × 2 tables and the chi‐square test in other cases.

For more material and methods data, see the Supporting Information section.

## 3 RESULTS

### 3.1 NCAPH overexpression induces mammary gland hyperplasia and breast cancer in mice

**FIGURE 1:**
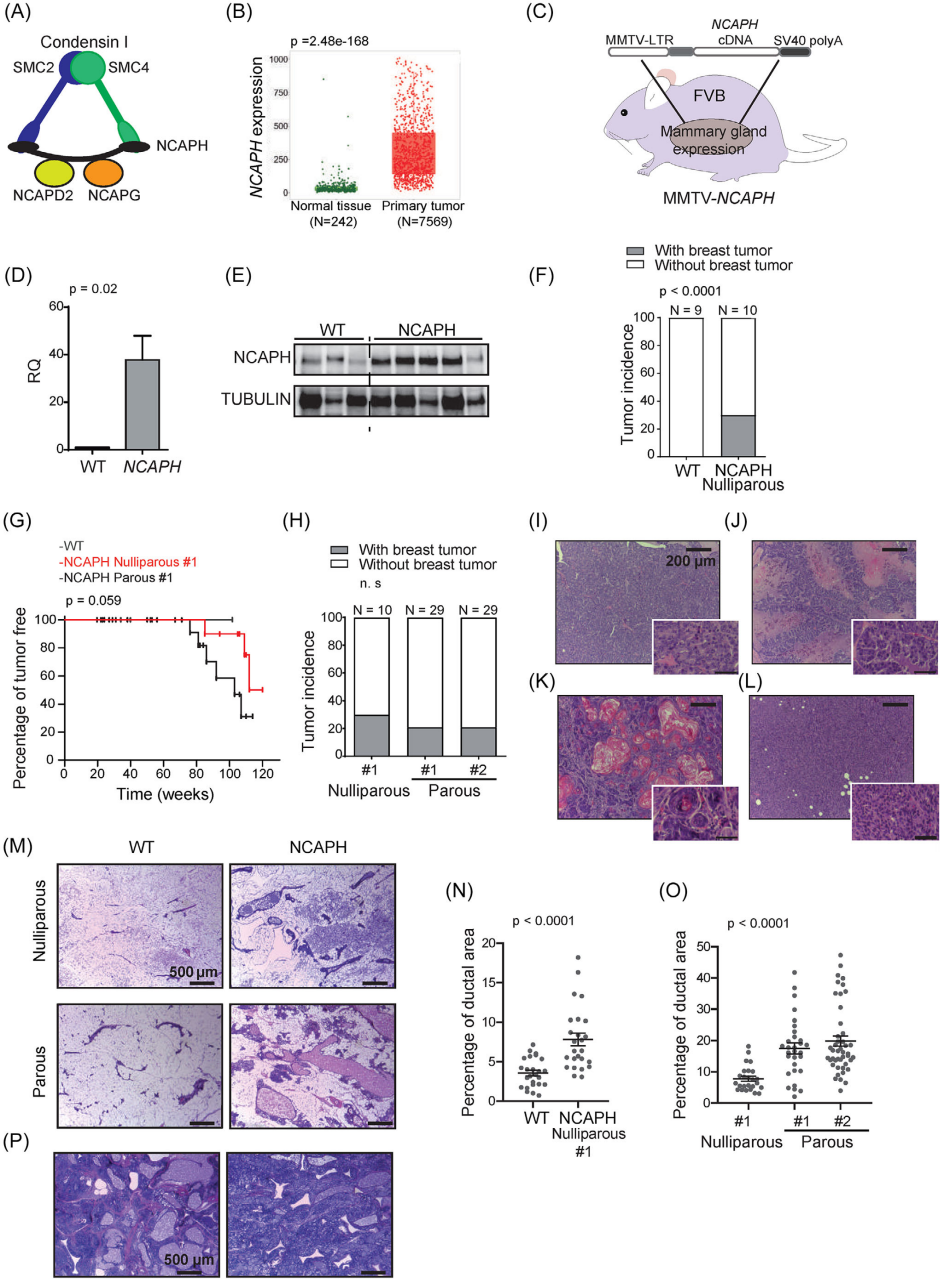
Transgenic mice that overexpress non‐SMC condensin I complex subunit H (NCAPH) develop breast cancer and mammary gland hyperplasias. (A) Condensin complex 1, of which NCAPH forms a part. (B) *NCAPH* is more strongly expressed in breast cancer than in normal mammary tissue. Analysis performed in the UALCAN portal using The Cancer Genome Atlas (TCGA) information. TPM, transcripts per million. (C) The figure shows *NCAPH* overexpressed under the Mouse Mammary Tumor Virus (MMTV) promoter to generate MMTV‐*NCAPH* transgenic mice. (D and E) Overexpression of *NCAPH* RNA in the mammary gland of transgenic mice quantified by qPCR (D) and the increase in protein seen in Western blots (E). (F) Incidence of breast cancer in mice overexpressing NCAPH after a 120‐week follow‐up. The controls were non‐transgenic mice with an FVB genetic background. (G) Tumour latency of MMTV‐*NCAPH*#1 nulliparous and parous mice. Kaplan–Meier curves and log‐rank test. (H) Incidence of breast cancer in parous mice overexpressing NCAPH after a 120‐week follow‐up. (I–L) Different histopathological patterns of breast cancer in MMTV‐*NCAPH* transgenic mice (Table S1). Glandular pattern (I), papillary pattern (J), squamous pattern with corneal pearls (K), solid pattern (L). (M) Ductal hyperplasia in nulliparous and multiparous MMTV‐*NCAPH* mice, with ductal dilation. (N and O) Increased ductal parietal area of the mammary gland in MMTV‐*NCAPH* nulliparous versus wild‐type mice (N). The multiparous mice had a larger ductal parietal area than the nulliparous mice (O). N and O, *t*‐test. (P) Detail of a mammary gland from two MMTV‐*NCAPH* mice with massive hyperplasia. SMC, structural chromosome maintenance.

NCAPH is a constituent of the condensin complex, which facilitates the assembly of interphase chromatin into chromosomes and orchestrates their segregation during mitosis and meiosis^21^ (Figure 1A). Initially, we found that NCAPH overexpression was associated with poor outcomes in breast cancer^49^, ^50^ (personal communications). This was determined through data mining analyses, specifically examining genes involved in mitosis and their correlation with adverse prognosis in breast cancer. We sourced our data from multiple databases: ETAM‐158,^51^ GSE1456,^52^ GSE2034,^53^ GSE4922^54^ and the dataset cited in Ref.^55^ (Figure S1A). Later, we identified an elevation in NCAPH levels in invasive ductal breast carcinomas compared to normal mammary tissues^56^ (Figure 1B). Consequently, this prompted us to investigate the potential involvement of NCAPH in the pathogenesis of breast cancer.

To determine whether NCAPH is a driver of breast cancer development, we generated transgenic mice overexpressing NCAPH under the control of the MMTV promoter (Figure 1C), inducing NCAPH overexpression in the mammary gland (Figure 1D,E). MMTV‐*NCAPH* nulliparous mice developed breast tumours during long‐term follow‐up, with 30% of mice developing breast cancer after 120 weeks (Figure 1F). Hence, the overexpression of *NCAPH* could drive breast cancer development. Oncogenes driven by the MMTV promoter are overexpressed during pregnancy because of promoter induction by gravidity hormones, resulting in more aggressive tumours.^57^ Indeed, the overexpression of NCAPH, regulated by the MMTV promoter in this transgenic line, resulted in breast tumour development with reduced latency post‐pregnancy, suggesting a dose‐dependent effect (Figure 1G). Both *MMTV‐NCAPH#1* and #2 mouse transgenic lines developed breast tumours after pregnancy, with no significant difference in tumour incidence (Figure 1H). Infiltrating ductal carcinomas generated by *NCAPH* overexpression had a range of histopathological features, including breast adenocarcinomas with papillary differentiation, squamous differentiation, or a mesenchymal pattern (Figure 1I **–**L and Table S1).

Interestingly, although most nulliparous *MMTV‐NCAPH* mice did not develop breast tumours after 2 years, they did show a significant increase in their ductal epithelial components. Indeed, *MMTV* promoter‐driven overexpression of *NCAPH* produced hypertrophic mammary glands with marked ductal hyperplasia. The ducts were formed by a normal, single row of epithelial cells with no atypia and a benign aspect (Figure 1M). However, there was a substantial increase in the number of ducts, increasing the total parietal ductal area (Figure 1N,O). Notably, two additional mice that were not included in the comparison exhibited massive ductal and stromal hyperplasia in the mammary gland and the absence of fatty tissue (Figure 1P). Together, these results indicate that NCAPH is oncogenic in breast tumours.

### 3.2 NCAPH expression is associated with poor outcome and response to therapy in luminal A breast cancer patients

**FIGURE 2:**
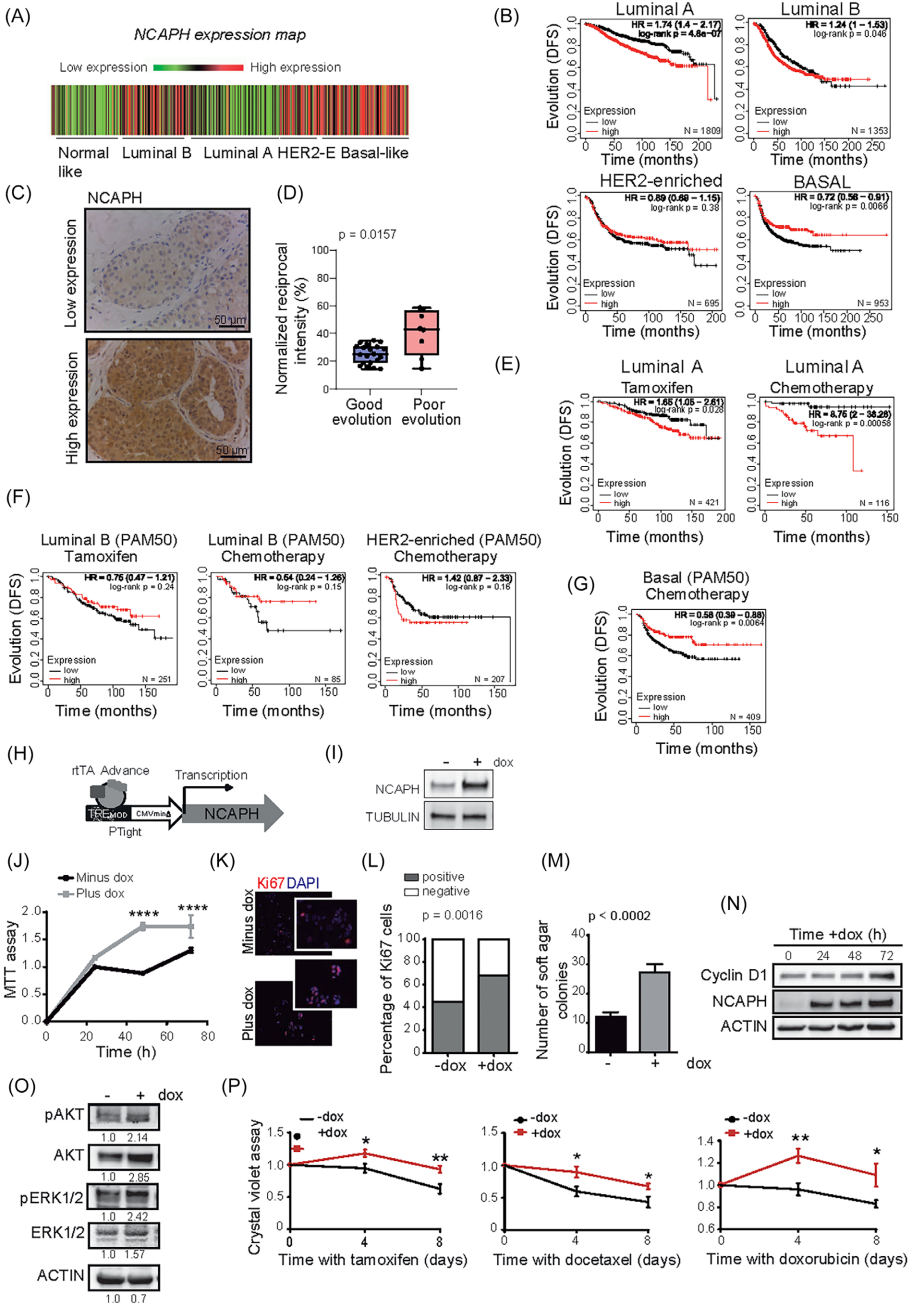
Intratumoural levels of non‐SMC condensin I complex subunit H (NCAPH) and outcome of breast cancer. (A) Representation of *NCAPH* expression in the various intrinsic subtypes of breast cancer. Data were obtained from the bc‐GenExMiner.^58^ (B) Outcome (disease‐free survival [DFS] probability) of patients with different intrinsic subtypes of breast cancer, as defined by Prediction Analysis of Microarray 50 (PAM50). The selection of patients for this panel does not consider the type of treatment received. (C) Analysis of NCAPH expression by immunohistochemistry (IHC) in luminal A tumours. The panel shows an example of strong and weak expressions. (D) Comparative analysis of NCAPH staining intensity in luminal tumours exhibiting good versus poor prognosis was performed using Fiji Is Just ImageJ (FIJI) software, adhering to Nguyen et al. methodology. Post‐colour deconvolution for DAB staining, reciprocal intensity (RI) in arbitrary units was determined by the difference in mean intensity between the epithelium and the background. These RI values, which inversely correlate with staining intensity, were normalised to the background intensity to ensure precise representation. (E) Outcome (DFS) in luminal A patients (as defined by PAM50) treated with tamoxifen or chemotherapy. (F) Patients with luminal B or HER2‐enriched tumours were treated with tamoxifen and chemotherapy. (G) Basal tumours (as defined by PAM50) were treated with chemotherapy. In (B), (F) and (G), the panels were generated with the Kaplan–Meier Plotter database, considering high and low intratumoural levels of *NCAPH* mRNA defined by the best cutoff.^59^ (H) Diagram of the TET on system used to express NCAPH in response to doxycycline (dox). (I) Expression of NCAPH in the MCF‐7 cell line after a 24 h induction with dox. (J) Assessment of cell proliferation in the MCF‐7 cell line following NCAPH induction using the MTT test. (K) Representative image of Ki‐67 staining (red) and DAPI (blue) after a 24 h induction with dox in the inducible MCF‐7 NCAPH cell line. (L) Quantitative analysis of proliferative cells positive for Ki67 using Fisher's exact test. (M) NCAPH induction led to an increase in the number of MCF‐7 colonies formed in soft agar. (N) A Western blot illustrates the upregulation of cyclin D1 post‐NCAPH induction. (O) Analysis of AKT and ERK signalling pathways in the MCF‐7 cell line post‐NCAPH induction as evidenced by Western blotting. Densitometric quantification of each protein is displayed below its respective band. (P) Increased cell resistance to various treatments, including tamoxifen, docetaxel and doxorubicin, observed after NCAPH induction. The number of patients included in each Kaplan–Meier curve panel is indicated within each respective graph. SMC, structural chromosome maintenance.

After demonstrating that NCAPH is involved in breast cancer development (Figure 1) and is linked to poor prognosis in breast cancer (Figure S1A), we aimed to identify the specific intrinsic subtype of breast cancer where intratumoural NCAPH levels are associated with unfavourable outcomes. Interestingly, the most aggressive subtypes of breast cancer—specifically basal and HER2‐enriched—exhibited the highest levels of NCAPH (Figure 2A).^58^ However, elevated NCAPH expression was also observed in patients from other subgroups. Consequently, we explored if NCAPH levels could differentiate between prognostically favourable and unfavourable forms within each intrinsic breast cancer subtype.

Breast cancer subtype classification and prognosis have been enhanced by gene signatures such as Prediction Analysis of Microarray 50 (PAM50).^9^ PAM50 provides a more precise intrinsic subtype classification than the St. Gallen approach, especially evident in the distinction between luminal A and B subtypes, underscoring its superior molecular accuracy.^60^ Consequently, we employed PAM50 to explore whether distinct evolutionary groups emerge from intratumoural NCAPH expression under optimal classification conditions.^59^

Although *NCAPH* expression was weak in the luminal A subtype compared with some other subtypes (Figure 2A), we found that patients with high levels of *NCAPH* RNA were associated with poor outcome (*p* = 4.8 × 10^−7^). Paradoxically, basal‐like tumours were associated with a good clinical outcome (*p* = .0066) (Figure 2B).

These results indicate the existence of a subpopulation of luminal A tumours that have poor outcome, which can be distinguished by their high intratumoural levels of *NCAPH*. We evaluated NCAPH levels by IHC in a cohort of patients with luminal A tumours (Table S2A), and again, we confirmed the higher NCAPH protein levels in patients with a poor outcome (presence of liver metastases) than in those who evolved well in the 10‐year follow‐up. The median RI for the good prognostic group was 25.06%, with an interquartile range (IQR) of [18.94–30.60]. For the poor prognostic group, the median RI was 43.06%, accompanied by an IQR of [24.74–56.44]. Across the entire cohort, the median RI was observed to be 28.73%, with an IQR of [19.45–34.40] (Figure 2C,D). Moreover, NCAPH levels were not associated with other tumour characteristics such as grade, stage, histological subtype, or Ki‐67 staining (Table S2B). Significantly, high levels of *NCAPH* in luminal A tumours were associated with poor response to endocrine therapy and chemotherapy in the long‐term follow‐up (Figure 2E). Thus, elevated *NCAPH* levels in luminal A tumours from patients who have received hormonal therapy or chemotherapy correlate with poor long‐term outcomes, thus serving as a prognostic marker. This was in contrast with other intrinsic tumour subtypes that responded to therapy independently of *NCAPH* levels (Figure 2F), except for basal tumours, which tended to respond well to chemotherapy when *NCAPH* levels were high (Figure 2G).

To ascertain that elevated levels of NCAPH were associated with a diminished response to chemotherapy, we engineered an MCF7 luminal A breast cancer cell line wherein NCAPH overexpression could be triggered by doxycycline (Figure 2H,I). The induction of NCAPH overexpression in MCF7 cells augmented their viability and proliferation (Figure 2J–L), which correlated with the generation of significantly more colonies on soft agar (Figure 2M) and elevated levels of cyclin D1 expression (Figure 2N). The elevated levels of NCAPH were correlated with an increase in both total and phosphorylated AKT levels, which play a pivotal role in regulating various cellular processes, including proliferation, survival and cell growth (Figure 2O). Remarkably, the upregulation of NCAPH led to partial resistance to therapy in MCF7 cells (Figure 2P). We observed no discernible differences in cell viability in the basal BT‐549 cell line following the induction of NCAPH expression or doxorubicin treatment (Figure S2A–C). Thus, high NCAPH expression in luminal A breast tumours is associated with a poorer prognosis and therapy response, indicating its potential as an identifier for high‐risk luminal A tumours.

### 3.3 Intratumoural levels of NCAPH are associated with poor outcome in luminal HER2^+^ tumours

**FIGURE 3:**
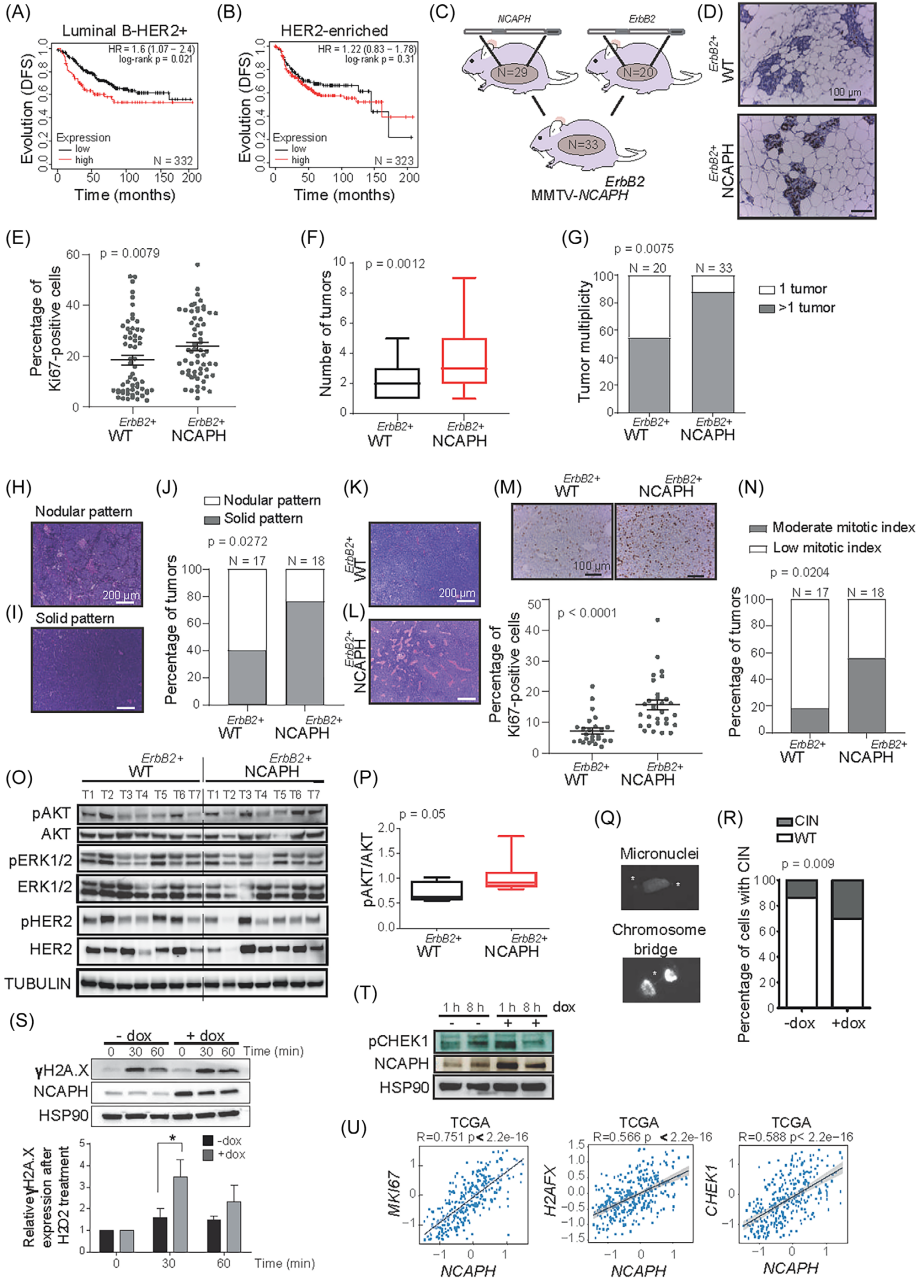
Effect of non‐SMC condensin I complex subunit H (NCAPH) expression on luminal ERBB2‐positive tumours in vivo. (A and B) Overexpression of *NCAPH* in patients with luminal ER‐positive HER2^+^ breast tumours was associated with a worse outcome (A), while this association was not seen in HER2^+^ ER‐negative tumours (B). Panels A and B were generated in the KM Plotter database.^59^ Each graph displaying a Kaplan‐Meier curve panel specifies the number of patients it encompasses. Outcome (disease‐free survival [DFS] probability). Analysis using the KM plotter's optimal cutoff. (C) Scheme to generate double‐transgenic mice Mouse Mammary Tumor Virus (MMTV)‐*NCAPH^ErBb2^*. (D and E) Immunohistochemical proliferation assessment in non‐tumoural mammary glands: analysis of mammary glands from MMTV‐*ErbB2* (upper panels) and MMTV‐*NCAPH^ErbB2^* double‐transgenic mice (D), with representative Ki67 immunohistochemistry images. Five mice per group were analysed, and field analysis results are shown in panel E, using the Mann–Whitney *U*‐test. (F and G) Tumour development quantification: double‐transgenic mice exhibited significantly more tumours (F) and higher tumour multiplicity (G) compared to MMTV‐*ErbB2* mice. (H–J) Histopathological pattern analysis: this section assesses different histopathological patterns in the samples, focusing on nodular (H) and solid patterns (I). Notably, the solid pattern occurred more frequently in double‐transgenic mice compared to MMTV‐*ErbB2* mice (J). Ten mice per group were evaluated with a chi‐squared test. (K and L) Vascularisation variability in mouse tumours: the panels compare vascularisation in tumours from MMTV‐*ErbB2* and MMTV‐*NCAPH^ErbB2^* double‐transgenic mice. Haematoxylin–eosin staining is used to emphasise vascular differences between MMTV‐*ErbB2* (panel K) and MMTV‐*NCAPH^ErbB2^* double‐transgenic mice (panel L). (M) Proliferative activity in tumour tissues: this section uses Ki67 immunohistochemical analysis to evaluate tumour proliferation in MMTV‐*ErbB2* (left) and MMTV‐*NCAPH^ErbB2^* double‐transgenic mice (right). Quantitative results from this analysis are displayed below the corresponding images. (N) Mitotic index quantification using chi‐squared test. (O and P) Analysis of ERBB2 downstream signalling molecules via Western blot (O) with pAKT quantification (P). (Q and R) NCAPH induction increased genomic instability, evidenced by more micronuclei and chromosomal bridges (CIN). Micronuclei and chromosomal bridge images (Q) are accompanied by CIN quantification (R), analysed with Fisher's exact test. (S) Genomic stress linked to increased NCAPH is shown by elevated ɤH2AX levels post‐H_2_O_2_ exposure and during 30‐ or 60‐min recovery. (T) pCHEK1 expression changes following NCAPH induction are displayed via Western blot. (U) RNA expression correlation in luminal breast cancer: this panel shows the correlation between *NCAPH* RNA and *Ki67*, *CHEK1* and *H2AX* in luminal breast cancer samples from The Cancer Genome Atlas (TCGA) database. It illustrates the correlation coefficients and patterns using CANCERTOOL.^62^ SMC, structural chromosome maintenance.

*NCAPH* levels were not associated with changes in the outcome of HER2‐enriched tumours, which are ER negative, as defined by the PAM50 (Figure 2A,E). Since luminal HER2 tumours are not defined as an intrinsic subtype of breast cancer defined by PAM50, immunohistochemical classification was used to define the role of NCAPH in the prognosis of this tumour subtype.^61^ Interestingly, high *NCAPH* levels were associated with poor clinical outcome of HER2^+^ luminal tumours (ER positive), confirming that *NCAPH* did not influence the outcome of HER2‐enriched tumours defined by IHC either (Figure 3A,B).

Thus, to investigate the role of NCAPH in the pathogenesis of luminal‐HER2^+^ tumours, we crossed MMTV*‐NCAPH* mice with MMTV‐*ErbB2* transgenic mice that developed luminal ERBB2 breast tumours^34^ (Figure 3C). An increase in epithelial proliferation was evident in the mammary glands of double‐transgenic MMTV‐*NCAPH^ErBb2^* mice relative to their MMTV*‐ErbB2* counterparts when Ki‐67^+^ cells were stained by IHC and counted (Figure 3D,E). Interestingly, the overexpression of *NCAPH^ErBb2^* in the mammary tissue of mice (MMTV‐*NCAPH^ErBb2^*) developed significantly more tumours than their MMTV*‐ErbB2* counterparts (Figure 3F,G), although no differences were observed in any other pathophenotypes of this disease.

Notably, while *NCAPH* transgenic mice developed distinct histopathological types of breast cancer (Figure 1J–M), the MMTV*‐NCAPH^ErBb2^* double‐transgenic mice developed only infiltrating ductal adenocarcinomas, suggesting that the *ErbB2* oncogene exerts a dominant effect on the tumour phenotype. Therefore, the *ErbB2* oncogene appears to reprogram tumour differentiation so that instead of the histopathologically distinct tumours induced by NCAPH overexpression, the characteristics of infiltrating ductal adenocarcinoma predominated. Moreover, the double‐transgenic mice predominantly developed infiltrating ductal adenocarcinomas exhibiting a solid histopathological pattern (Figure 3H–J), enhanced vascularisation, and significantly heightened tumour proliferation compared to their MMTV‐*ErbB2* counterparts (Figure 3K–M). Remarkably, tumours induced by NCAPH overexpression demonstrated a higher mitotic index (Figure 3N).

The different histopathological behaviors of tumours from MMTV*‐NCAPH^ErbB2^* double‐transgenic mice prompted us to study molecules from some of the main pathways downstream of ERBB2. Intratumoural signalling was evaluated by assessing pAKT/pmTOR and pERK in Western blots of seven tumours from each phenotype, and only pAKT levels were significantly higher in tumours from the double‐transgenic mice than in those from the single‐transgenic mice (Figure 3O,P). No significant differences were observed in the pERBB2 and ERBB2 levels between tumours from MMTV‐*ErbB2* mice and double‐transgenic MMTV‐ *Ncaph^ErbB2^* mice (Figures 3O and S3A,B), ruling out their involvement in the differing tumour behaviors of the two groups. Additionally, the tumours from the double‐transgenic MMTV‐*Ncaph^ErbB2^* mice exhibited higher basal apoptosis, as indicated by elevated levels of cleaved Caspase 3 (Figure S3C).

It is noteworthy that NCAPH overexpression in MCF7 cells also elevated the proportion of cells exhibiting chromosomal instability (CIN), wherein micronuclei and chromosomal bridges were identifiable (Figure 3Q,R). Genomic instability triggers genomic stress,^63^, ^64^ as evidenced by the escalated levels of ɤH2AX and pCHEK1 following NCAPH overexpression and exposure to H_2_O_2_ (Figure 3S,T). In human tumours, a robust positive correlation was discerned between NCAPH expression and proliferation markers (Ki67) or genomic instability markers (H2AX and CHEK1) (Figure 3U).

The unique histopathological characteristics observed may emanate from elevated levels of NCAPH in the mammary gland, thereby exacerbating genomic instability and instigating secondary oncogenic events that target diverse tumour differentiation pathways.^65^

Together, these findings propose that NCAPH overexpression in ERBB2 tumours engenders more aggressive histopathology, solid tumours with high mitotic indices, and enhanced vascularisation and cell proliferation. Thus, elevated levels of NCAPH promote a more aggressive form of breast cancer, potentially elucidating the adverse progression observed in luminal tumours.

### 3.4 NCAPH expression is associated with poor breast cancer outcome in a genetically heterogeneous cohort of mice

**FIGURE 4:**
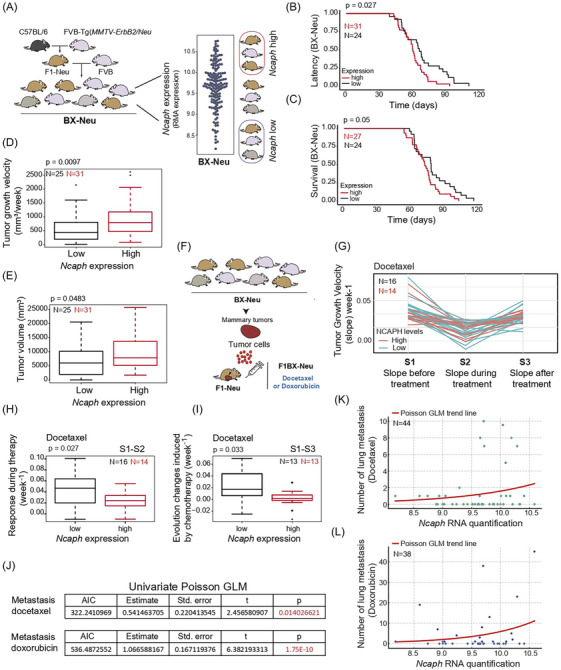
Intratumoural levels of *Ncaph* were associated with a worse outcome and response to chemotherapy in a cohort of mice generated by backcrossing (BX‐*Neu^+^*). (A) Generation scheme for BX‐*Neu^+^* cohort: the BX‐*Neu^+^* cohort was created by backcrossing transgenic Mouse Mammary Tumor Virus (MMTV)‐*ErbB2* mice (FVB/N background) with C57BL/6 mice, which are resistant to breast cancer. The first‐generation offspring (F1) were further crossed with wild‐type FVB/N mice, producing the BX‐*Neu^+^* cohort. This cohort is genetically diverse, displaying a range of phenotypic traits and intratumoural *Ncaph* gene expression. Mice from the highest and lowest tertiles of *Ncaph* expression were selected for evaluation, as shown in the schema. Gene expression was measured using robust multiarray analysis (RMA) log2. (B and C) Decrease in tumour latency probability (B) and survival probability (C) in BX‐*Neu^+^* mice with elevated intratumoural levels of *Ncaph*. (D and E) Tumour growth velocity and final volume for tumours with high or low levels of *Ncaph*. Weekly tumour volume was measured in mm^3^ using a digital caliper and calculated as (greater diameter × lesser diameter^2^)/2. Growth velocity was determined by (final volume – initial volume)/illness duration in weeks, expressed in mm^3^/week. (F) Diagram showing the allogeneic transplantation strategy, passing tumours generated in BX‐*Neu^+^* mice into immunocompetent F1 mice. Assessments were conducted on mice selected from the top and bottom tertiles. (G–I) Docetaxel Impact on Tumour Growth Relative to Ncaph Levels. (G) This panel displays growth curve slopes at crucial docetaxel treatment stages: pre‐chemotherapy (S1) and during chemotherapy (S2, indicating peak response). Response during treatment (RDT) is derived as S1 minus S2, with a higher RDT signifying better response. (H) RDT comparison across tumours with different Ncaph levels, classified by external tertiles. (I) Post‐chemotherapy growth changes or Evolution Changes Induced by Chemotherapy (ECIC) are shown by comparing slopes pre‐chemotherapy (S1) and post‐chemotherapy (S3), with a greater ECIC value indicating improved treatment outcome (external tertiles). (J) Univariate Poisson regression analysis indicates a positive correlation between intratumoural *Ncaph* levels and post‐chemotherapy lung metastases, with higher *Ncaph* levels linked to increased metastases (*p*‐values < .05). A positive ‘Estimate’ indicates a correlation between higher Ncaph levels and an increased count of lung metastases. (K and L) Poisson GLM analysis further confirms this correlation in mice treated with docetaxel (K) and doxorubicin (L), where individual mouse data points display the relationship between *Ncaph* RNA levels and metastasis count. The model's red line illustrates the trend, underscoring *Ncaph's* role in lung metastasis after breast tumour treatment with these drugs.

Genetically heterogeneous mouse cohorts better reflect the heterogeneity observed in the human population, facilitating the identification of the genetic and transcriptomic determinants associated with disease outcome.^35^, ^66^, ^67^ Thus, to analyse the contribution of NCAPH expression to the heterogeneous outcome of luminal ERBB2 breast cancer, we used a genetically heterogeneous cohort of MMTV‐*ErbB2* transgenic mice generated by backcrossing (BX‐*Neu^+^* mice after that) (Figure 4A). We crossed MMTV*‐ErbB2* transgenic mice, which are on a susceptible FVB genetic background, with non‐transgenic mice on a C57BL/6 genetic background, which is resistant to breast cancer development. We generated F1C57BL6/FVB MMTV‐*ErbB2* mice (F1‐*Neu^+^* mice hereafter) that were backcrossed with FVB mice to generate BX‐*Neu^+^* mice, a cohort of mice with more varied breast cancer outcome than genetically homogenous mouse strains.

This BX*‐Neu^+^* cohort was used to evaluate whether intratumoural levels of *Ncaph* were associated with heterogeneous breast cancer outcome, demonstrating that high intratumoural *Ncaph* RNA levels were associated with shorter tumour latency and survival (Figure 4B,C), faster tumour growth, and larger tumour volume (Figure 4D,E). Thus, high levels of *Ncaph* are associated with poor outcome of luminal *ErbB2* breast cancer in the cohort of BX‐*Neu^+^* mice.

Since elevated *NCAPH* levels mediate a poor response to chemotherapy in breast cancer patients and cells (Figure 1G,L–N), we evaluated the response of tumours generated in BX‐*Neu* ^+^ mice to chemotherapy. Following the laws of transplantation in mice,^68^ breast cancer tumours that developed in the backcross cohort were transplanted into F1 mice, and their responses to anthracycline and taxane chemotherapy were evaluated (Figure 4F). This strategy allowed us to evaluate the response of breast cancer to chemotherapy in an extracellular context as homogeneously as possible. Thus, differences in treatment responses can be primarily attributed to differences at the cell‐autonomous level. Tumours with high levels of *Ncaph* responded worse to docetaxel treatment, as reflected by a smaller reduction in tumour size (Figure 4G,H). In addition, the growth rate of tumours with high levels of *Ncaph* was faster, and their outcome was worse after chemotherapy than those that expressed *Ncaph* more weakly (Figure 4G,I). However, *Ncaph* levels did not appear to influence the local response to doxorubicin (Figure S4A–C). After chemotherapy with either doxorubicin or docetaxel, lung metastases were most evident in mice with high intratumoural *Ncaph* levels (Figures 4J–L and S4D,E).

These findings suggest that high intratumoural levels of NCAPH are associated with resistance to breast cancer chemotherapy.

### 3.5 A gene signature based on NCAPH expression defines poor tumour outcome in mice and humans

**FIGURE 5:**
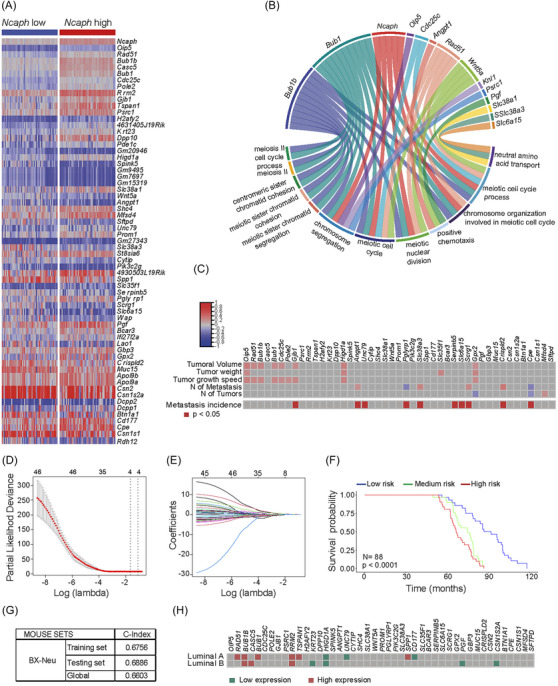
Identifying a gene signature associated with non‐SMC condensin I complex subunit H (*NCAPH*) in the Mouse Mammary Tumor Virus (MMTV)‐*ErbB2* cohort of mice generated by backcrossing (BX‐*Neu^+^*) recognises the poor outcome in mice and humans. (A) Heatmap showing the 64 differentially expressed genes between tumours with high or low intratumoural levels of *Ncaph* in BX‐*Neu^+^* mice, of which 45 were shared with humans. Extreme tertiles of *Ncaph* expression were taken to define the high and low levels of *Ncaph*. The criteria for underexpressed and overexpressed genes were a ← twofold and a > twofold change, respectively, and a value of *p* < .05 (Table S3). (B) Gene Ontology analysis shows the main biological functions in which the 45 genes of the signature participate (Table S4). (C) Some of the genes in the 45‐gene signature were associated with the poor outcome of breast cancer in MMTV‐*ErbB2* mice generated by backcrossing. Correlation of the intratumoural levels of these genes with specific pathophenotypes of breast cancer in BX‐*Neu^+^* mice: Pearson's test. The incidence of metastasis was evaluated with a chi‐squared test. (D–G) Least absolute shrinkage and selection operator (LASSO) regression model that defines poor outcome in BX‐*Neu^+^* mice. The regression coefficient map of genes in the LASSO model, using cross‐validation to select the optimal tuning parameter (*λ*). The dotted vertical lines were drawn at the optimal values using the minimum criteria and 1 standard error (SE) of the minimum criteria (the 1 – SE criteria) (D). The graph shows the screening path of the LASSO regression model. Each curve represents a LASSO coefficient of the 45 prognostic genes, and the x‐axis indicates the regularisation penalty parameter. When the number of variables was 4, the partial likelihood deviation was at the minimum, corresponding to the minimum *λ* value (E). The LASSO regression model generated differentiates between low‐, intermediate‐ and high‐risk BX‐*Neu^+^* mice in terms of survival probability and as defined by tertiles. Kaplan‒Meier curve and log‐rank test (F). Goodness‐of‐fit measures for the generated LASSO models in the BX‐*Neu^+^* mouse cohort. Training, testing and global model results (G). (H) The heatmap represents the association of gene transcript levels with the prognosis of human luminal breast tumours, in relation to *Ncaph* levels in mouse breast tumours from the backcross cohort. Colour coding: Brown indicates genes where high transcript levels are statistically significantly associated with poor prognosis; green denotes genes where low transcript levels correlate with poor prognosis. The heatmap is supported by detailed data on patient count, expression values, and *p*‐values in Table S6. SMC, structural chromosome maintenance.

We examined the transcriptomic context in which *Ncaph* expression was associated with poor breast cancer outcome. The wide range of breast cancer outcome in backcross mice^35^, ^63^ and the diverse expected patterns of gene expression^67^ (Figure 4) make this cohort an excellent tool for identifying transcripts associated with high levels of *Ncaph* expression and poor breast cancer outcome.^35^, ^69^ We identified 64 transcripts associated with high intratumoural *Ncaph* levels in breast tumours from the backcross cohort, of which 45 were shared with humans (Figures 5A and S5A,B and Table S3). The functions of this 45‐gene signature were assessed using GO enrichment analysis.

Unsurprisingly, several genes associated with high *Ncaph* levels were involved in processes related to correct condensation and segregation of chromosomes during mitosis (Figures 5B and S5C and Table S4), and some were also correlated with poor breast cancer outcome in the BX‐*Neu* ^+^ cohort of mice (Figure 5C). When we integrated several of these genes into a multivariate LASSO regression model to define poor tumour outcome in the BX‐*Neu* ^+^ mouse cohort (Figure 5D,E and Table S5), four genes were identified that were associated with poor survival in BX‐*Neu* ^+^ mice: *Oip5*, *Higd1a*, *Shc4* and *Scrg1* (Figure S5D–F). Interestingly, the intratumoural levels of certain genes identified in the heterogeneous BX‐*Neu^+^* model, and associated with elevated levels of *Ncaph*, also correlated with adverse clinical outcomes (RFS) in patients with the intrinsic luminal A subtype of breast cancer^59^ (Figure 5H and Table S6).

In conclusion, elevated expression of *NCAPH* was linked to a set of gene transcripts. The intratumoural levels of these transcripts, akin to *NCAPH* itself, were also associated with the adverse progression of luminal breast cancer in both mice and humans.

### 3.6 Identification of a genetic model associated with poor outcome in patients with luminal tumours

Despite having the best overall prognosis among the intrinsic subtypes, luminal A tumours display significant variation in prognosis. It is crucial to identify patients with poor prognoses for improved survival via initial therapeutic enhancements. High levels of *NCAPH* were associated with poor outcome, especially in luminal A tumours (Figure 1D). Our study identified an array of genes that exhibited a notable correlation with elevated intratumoural levels of *Ncaph*, as depicted in Figure 5. We also found that some of these genes were associated with poor RFS in humans (Figure 5H). Therefore, we used a penalised multivariate LASSO regression model to identify a gene signature that reflects the poor prognosis of luminal A tumours.^31^

**FIGURE 6:**
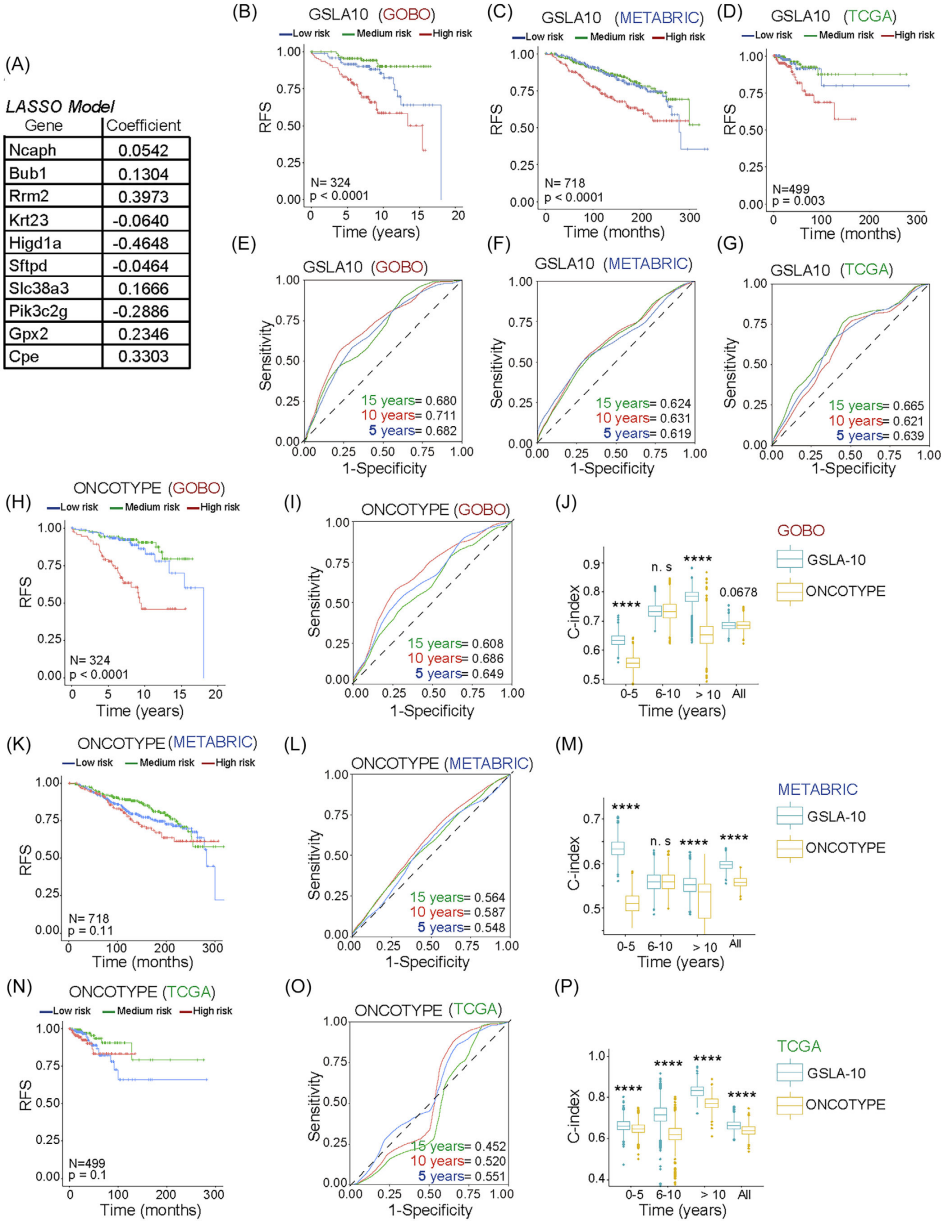
Comparative evaluation of Gene Signature for Luminal A 10 (GSLA10) and the Oncotype signature for prognostic determination in luminal A tumours. Study and validation of GSLA10 in patients on the Gene Expression‐based Outcome for Breast Cancer Online (GOBO), Molecular Taxonomy of Breast Cancer International Consortium (METABRIC) and The Cancer Genome Atlas (TCGA) datasets. (A) Hazard ratio illustration of each GSLA10 gene's individual risk contribution, as implicated in the least absolute shrinkage and selection operator (LASSO) model from the GOBO cohort. (B) Assessment of the GSLA10 model's capability in delineating luminal A tumour progression via Kaplan‒Meier curves from the GOBO cohort. (C) Validation of the robustness of the GSLA10 model for luminal A tumour progression definition using Kaplan‒Meier curves in the METABRIC cohort. (D) Validation of the Oncotype model's robustness in characterising luminal A tumour progression with Kaplan‒Meier curves from the METABRIC cohort. (E) Receiver operating characteristic (ROC) curves illustrating the prognostic prediction prowess of the GSLA10 model within the GOBO cohort. (F) ROC curves demonstrating the GSLA10 model's prognostic prediction in the METABRIC cohort. (G) ROC curves of the GSLA10 model for prognosis prediction in the METABRIC cohort. (H) The Oncotype model's efficacy in defining luminal A tumour outcome was evaluated with Kaplan‒Meier curves in the GOBO cohort. (I) ROC curves showcasing the Oncotype model's ability to predict prognosis within the GOBO cohort. (J) *C*‐index comparison for prognostic prediction between GSLA10 and Oncotype models within the GOBO cohort. (K) Validation of the Oncotype model's robustness in characterising luminal A tumour progression using Kaplan‒Meier curves in the METABRIC cohort. (L) ROC curves of the Oncotype model for prognostic prediction in the METABRIC cohort. (M) Comparison of the *C*‐index for prognostic prediction between the GSLA10 and Oncotype models in the METABRIC cohort. (N) Further validation of the Oncotype model's robustness in defining luminal A tumour progression, as illustrated by Kaplan‒Meier curves from the METABRIC cohort. (O) ROC curves of the Oncotype model for prognostic prediction in the METABRIC cohort. (P) Comparative evaluation of the *C*‐index for prognostic prediction between the GSLA10 and Oncotype models in the METABRIC cohort. Each graph in the Kaplan–Meier curve panels displays the number of patients it encompasses.

The LASSO regression model was generated from the GOBO database of 401 patients with luminal A‐diagnosed breast cancers.^41^ This cohort was divided into a training set (70%) and a test set comprising the remaining 30% to generate a polygenic risk score. First, bivariate analyses of the training set using Cox regression identified genes associated with poor outcome in terms of RFS (Table S7). Later, genes with a *p‐*value <.25 were used to generate a polygenic risk score using the restrictive LASSO regression model.^31^ Thus, the LASSO model was developed, and ten genes were identified that define disease prognosis, which we referred to as the GSLA10 (Figures 6A and S6A,B and Table S8). The prognoses in the training and testing sets and the global model were evaluated using the *C*‐index (Figure S6C,D).

GSLA10 discriminated between low‐risk (bottom 1/3 risk score), medium‐risk (middle 1/3 risk score) and high‐risk (top 1/3 risk score) RFS in patients (Figure 6B). We validated our model in two independent patient cohorts, METABRIC and TCGA‐BRCA, with 718 and 499 luminal A breast cancer cases, respectively. The *C*‐index, area under the curve (AUC) of the ROC curve, and log‐rank *p*‐value of the KM analysis were assessed when applying GSLA10 to the METABRIC and TCGA‐BRCA cohorts, confirming the predictive capability of GSLA10 (Figures 6C,D and S6D).

Additionally, we compared the ability of these signatures to define the prognosis of luminal A tumours in terms of RFS at different time points, evaluating the AUC for the first five years after diagnosis, between 5 and 10 years, and after 10 years in METABRIC and TCGA‐BRCA (Figure 6E–G). The ROC curves indicated that GSLA10 can predict the risk of relapse in these patients at different time points.

In addition, we constructed a risk model (Oncotype) based on 21 genes in the Oncotype. Each Oncotype gene's expression level was adjusted in relation to a set of five reference genes, as detailed in Paik et al.^6^ We provided a comprehensive comparison between GSLA10 and the Oncotype in terms of patient risk stratification and prognostic power in both the training cohort (GOBO) (Figure 6H–J) and the independent validation cohorts (METABRIC and TCGA‐BRCA) (Figures 6K–M,N–P and S6E). The comparison further confirmed the robustness and superior prognostic power of GSLA10 over Oncotype in luminal A tumours.

**FIGURE 7:**
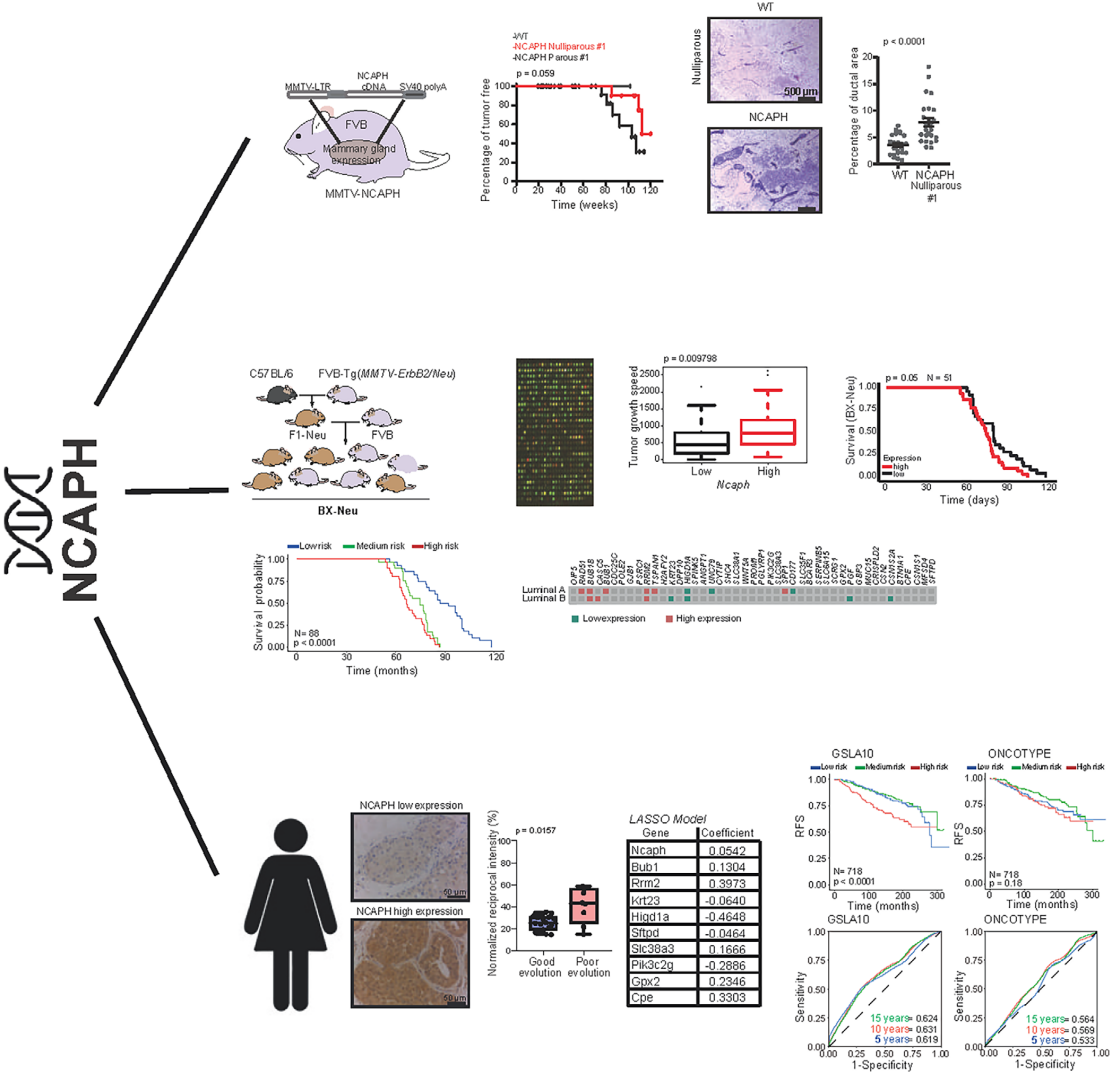
Graphical representation of non‐SMC condensin I complex subunit H's (NCAPH's) role in luminal A breast cancer progression. Using transgenic mouse models and a genetically diverse backcrossed mouse cohort, elevated NCAPH expression was linked to unfavourable tumour development. A heatmap showcases intratumoural NCAPH expression, correlating with poor prognosis. Least absolute shrinkage and selection operator (LASSO) analysis identified a 10‐gene signature, Gene Signature for Luminal A 10 (GSLA10), presented alongside its superior predictive capability compared to Oncotype DX, suggesting its potential in guiding personalised luminal A breast cancer treatments. SMC, structural chromosome maintenance.

In conclusion, the GSLA10 signature was associated with poor prognosis in luminal A patients. This model could help assess the prognosis of luminal A tumours and thus favor more personalised follow‐up and therapy for patients with breast cancer (Figure 7).

## 4 DISCUSSION

In our study, we discovered a gene signature (GSLA10) linked to high intratumoural NCAPH levels and the unfavourable progression of luminal A breast tumours. The need for accurate identification of the prognosis of this tumour type is imperative because of differing initial treatment responses, including the potential inclusion of chemotherapy.^70^, ^71^, ^72^, ^73^ Gene signatures, notably Oncotype DX, have been employed to identify potential chemotherapy beneficiaries among patients with ER‐positive tumours.^6^, ^74^, ^75^, ^76^ Moreover, luminal A tumours exhibit the highest post‐10‐year relapse risk despite endocrine therapy, necessitating precise patient identification and potential extended hormonal treatment.^77^, ^78^, ^79^ Consequently, enhancing the prognostic precision of luminal A tumours is of crucial importance. In this context, our GSLA10 gene signature has shown superior prognostic power over Oncotype DX in both short‐ and long‐term scenarios, suggesting its potential for tailoring luminal A patient treatment. Although GSLA10 demonstrated increased efficacy in predicting luminal A tumour prognosis, further studies are required to confirm whether this signature can reliably identify patients who may benefit from chemotherapy at diagnosis or from prolonged hormonal treatment.

We found that the overexpression of *NCAPH* is associated with poor prognosis, specifically in luminal A tumours and HER2^+^ luminal tumours. The specific association between high NCAPH levels and poor prognosis in luminal tumours may be related to the ability of condensin I complex to bind to ER enhancers. Indeed, condensins play an essential role in activating the expression of estrogen target genes through their activity at the transcriptional level.^80^ Moreover, the putative potentiation of estrogen signalling by NCAPH helps explain the hyperplasia observed in transgenic mice overexpressing NCAPH and their increased susceptibility to breast cancer development. This could also explain why NCAPH levels are not associated with the poor prognosis of breast tumours that do not express ER, such as HER2‐enriched and basal tumours, as well as the participation of NCAPH in the poor clinical outcome of other hormone‐dependent tumours, such as ovarian, endometrial, cervical and possibly prostate cancers.^81^, ^82^, ^83^, ^84^, ^85^, ^86^ Furthermore, luminal A tumours with worse outcome are thought to have higher genomic instability, alterations to P53^19^ and overexpression of genes that regulate mitosis.^20^

In the last 5 years, high levels of NCAPH expression have been associated with the pathogenesis and prognosis of several tumour types,^81^, ^82^, ^84^, ^86^, ^87^, ^88^, ^89^, ^90^, ^91^ including hepatocarcinoma,^92^ lung tumours,^93^, ^94^, ^95^ melanoma^96^ and endometrial cancer.^83^ During the development of this project, two studies on NCAPH in breast cancer were published. The first study demonstrated that in vitro, NCAPH expression is elevated in MCF7 cells compared to the non‐tumourigenic breast cell line, MCF10A.^22^ The second study revealed that downregulating NCAPH in MCF7 cells leads to a reduction in proliferation.^47^ In the present work, we substantially broaden the research on NCAPH's role in breast cancer development. In vitro, we have demonstrated its involvement in viability, proliferation, increased genomic instability, alterations in cellular signalling at various levels and resistance to multiple treatment modalities. Furthermore, we have introduced an in vivo transgenic mouse model that overexpresses NCAPH for the first time. Our findings suggest that NCAPH overexpression can eventually act as a primary oncogenic trigger for breast cancer. Notably, the overexpression of NCAPH leads to an enlargement of the non‐tumourous breast's glandular component, a recognised factor predisposing to breast cancer.^97^

Paradoxically, elevated NCAPH levels were associated with good evolution of basal tumours. Notably, NCAPH depletion is associated with inhibition of proliferation, migration and xenograft tumour formation in colon cancer cell lines. However, elevated NCAPH levels in patients with tumours have been associated with a better prognosis and survival rate than in patients with low levels of NCAPH.^89^ Similar results have been reported for human papillomavirus‐positive cervical cancer.^86^ For both tumour types, these features have been related to the enhanced proliferation induced by NCAPH, which might sensitise tumour cells to chemotherapy and radiation therapy.^86^, ^89^

Higher NCAPH levels linked to better outcomes in some cancers might potentially be due to their role in triggering oncogene‐induced apoptosis or senescence, a phenomenon also observed in other mitosis‐regulating genes such as Aurora A, Aurora B, Polo‐Kinase‐1, Cyc E and CDC25.^98^, ^99^, ^100^, ^101^ These genes' effects can vary, leading to either positive or negative outcomes, depending on how they interact with other oncogenic changes, such as p53 status.^101^ Moreover, increased cell proliferation and apoptosis have been noted with the overexpression of certain oncogenes like *MYC*, *E2A* and *E2F1*. Blocking apoptosis or senescence pathways in these cases allows cells to better tolerate high molecular levels and the resulting increased proliferation.^102^, ^103^, ^104^ While further investigation into the tolerance of elevated NCAPH levels and its interaction with other secondary oncogenic events is beyond the scope of our current study, the increased apoptosis or senescence triggered by NCAPH, due to enhanced proliferation and genomic stress, might explain our findings. Specifically, we observed a marginally reduced tumour incidence in instances where NCAPH is induced as a primary oncogenic event in normal breast tissue, as compared to non‐induced cases. This was evident in MMTV‐*Ncaph* multiparous mice in contrast to their nulliparous counterparts. Additionally, a higher rate of apoptosis was noted in MMTV‐*Ncaph^ErbB2^* double‐transgenic tumours than in single‐transgenic MMTV‐*ErbB2* tumours. In these cases, although higher levels of *Ncaph* were associated with increased tumour proliferation, the apoptosis (and potentially senescence) induced by Ncaph and the oncogenic stress it causes were still observable.

Our study also reveals a correlation between NCAPH and poor outcomes in HER2‐positive luminal tumours in both humans and mice. In mice, this association results in breasts having an expanded glandular component and heightened proliferation both of which are recognised as breast cancer risk factors.^105^, ^106^, ^107^ It was evident that HER2^+^ luminal tumours overexpressing *NCAPH* are more aggressive, characterised by rapid growth, increased proliferation and a high mitotic index. Collectively, our discoveries substantially augment the current understanding of NCAPH's role in the onset and progression of breast cancer.

Given our findings that elevated NCAPH levels correlate with poorer outcomes in luminal ERBB2 tumours in transgenic mice, we identified a gene signature associated with high NCAPH levels. This was also conducted using a backcross cohort of mice with luminal ERBB2 tumours, as they epitomise an extension of the phenotypic presentation of breast cancer and its associated transcriptomics.^50^ In backcross models, a notable phenotypic variation in breast cancer is observed alongside a heightened transcriptomic variation, facilitating the association of both phenotype and transcriptome with their genetic regulation.^64^, ^66^, ^69^ Genetically heterogeneous mouse cohorts better reflect the heterogeneity observed in the human population, facilitating the identification of the genetic and transcriptomic determinants associated with disease outcome.^35^, ^66^, ^67^ In this study, we utilised genetically heterogeneous mouse cohorts as a model system. This approach is particularly relevant because it mirrors the genetic diversity inherent in the human population. Such heterogeneity is a crucial factor in biomedical research, as it allows for a more accurate representation of the complexities and variabilities observed in human diseases. This modelling is especially significant in the context of identifying genetic and transcriptomic determinants that drive disease outcome. Studies have shown that the use of genetically uniform models often fails to capture the full spectrum of disease manifestations.^35^, ^66^, ^67^ In contrast, heterogeneous cohorts provide a broader genetic context, which is instrumental in uncovering the multifaceted nature of gene–disease interactions. This diversity enables the identification of subtler genetic variants and transcriptomic profiles that might be overlooked in more genetically homogeneous models. Enrichment analyses revealed that some of the identified genes govern cell cycle progression and mitosis.

Interestingly, some of these genes were also associated with poor breast cancer outcome in BX‐*Neu* ^+^ mice, both individually and after the application of the LASSO regression model. It is important to note that some genes in this signature were individually associated with poor outcome of intrinsic luminal A subtypes in humans in the KM plotter database.^59^ Moreover, the application of some of these genes in a multivariate LASSO regression model identified luminal A tumours that relapsed in three human breast cancer datasets. It is plausible that patients with luminal A tumours, exhibiting high levels of NCAPH and displaying the associated signature could represent a distinct subgroup of tumours. This subgroup might exhibit an intermediate prognosis, positioned between those observed for the luminal A and luminal B subtypes. Further research is required to investigate this hypothesis and elucidate its potential taxonomic implications.

Our encouraging findings with GSLA10 indicate the potential value of conducting future prospective studies. These studies should assess the efficacy of GSLA10 both independently and in combination with other signature biomarkers like OncotypeDX, specifically evaluating their impact on therapeutic decision making and patient outcomes.

## 5 CONCLUSIONS

In conclusion, we have demonstrated that NCAPH is involved in the pathogenesis of breast cancer. Moreover, an NCAPH‐associated signature defines the outcome of the luminal A breast cancer subtype. The potential of GSLA10 to distinguish a specific cohort of patients with luminal A tumours, who might benefit significantly from intensified treatment protocols aimed at preventing relapse, is indeed stimulating. Certainly, the GSLA10 signature could serve as a diagnostic tool to identify patients with luminal A tumours who are at risk of poor prognosis. This insight may enable healthcare providers to devise personalised treatment strategies that are more efficacious for these patients.

## AUTHOR CONTRIBUTIONS

Marina Mendiburu‐Eliçabe contributed to the study, generation of LASSO models and statistical analyses of the mouse and patient databases. He also critically reviewed the manuscript. Natalia García‐Sancha and Roberto Corchado‐Cobos have been used in studies involving transgenic mice, including generation of mouse lines, necropsies, tissue analysis, histopathology and IHC. Both provided a critical review of the manuscript. Angélica Martínez‐López studies have been conducted on cell lines, encompassing the generation of stable lines, cultures, feasibility analysis, proliferation and signalling pathways. He critically reviewed the manuscript. Hang Chang and Jian Hua Mao engaged in human database studies comparing GSLA10 with Oncotype DX and supervised the statistical analyses of the manuscript. Adrián Blanco‐Gómez analysed mice produced by backcrossing and studied allogeneic transplantation and chemotherapy treatments. Ana García‐Casas, Andrés Castellanos‐Martín and Nélida Salvador contributed to in vitro studies, generation of stable cell lines and signalling pathway analysis. Andrés Castellanos‐Martín specifically focuses on the generation and analysis of mice produced by backcrossing. Alejandro Jiménez‐Navas was involved in revising the survival curves and quantifying human IHC studies using software. Manuel Jesús Pérez‐Baena participated in in vitro studies on stable triple‐negative breast cancer cells. Manuel Adolfo Sánchez‐Martín contributed to the generation of transgenic mice and microinjection and chimera analyses. María Del Mar Abad‐Hernández and Sofía Del Carmen, both pathologists, evaluated the histopathology and IHC samples of luminal A human breast tumours. Juncal Claros‐Ampuero, Juan Jesús Cruz‐Hernández and César Augusto Rodríguez‐Sánchez are oncologists who contributed to the diagnosis and clinical follow‐up of breast cancer patients, and their tumours were analysed using IHC in this study. María Begoña García‐Cenador and Francisco Javier García‐Criado provided logistic support, focusing on animal maintenance and development of surgical protocols for breast tumour interventions. Rodrigo Santamaría Vicente conducted the preliminary bioinformatics studies. Sonia Castillo‐Lluva and Jesús Pérez‐Losada played pivotal roles in the project's conception, supervision and administration. They secured funding, managed the project and were actively involved in writing, editing and critical review of the manuscript. Their contributions also spanned the study and analysis of transgenic mice, and the conceptualisation and revision of statistical data.

## CONFLICT OF INTEREST STATEMENT

The authors declare they have no conflicts of interest.

## ETHICS STATEMENT

All the mice were housed at the Animal Research Facility of the University of Salamanca. All procedures were approved by the Institutional Animal Care and Bioethics Committee of the University of Salamanca. Committee reference number: 197‐2017. Human primary breast tumours were collected at the University Hospital of Salamanca (Salamanca, Spain), after the hospital's Institutional Ethics Review Board approved the protocols for collecting and using patient samples. Written informed consent was obtained from all patients to conduct the study on these tumour samples. Committee reference number: PI 2019 12 396.

## CONSENT FOR PUBLICATION

The Consent for Publication statement is satisfactory and in compliance with the required ethical standards. This assurance is based on the fact that our study does not include any photographs or data that could potentially reveal the identity of the participants involved. We have taken diligent care to ensure that all personal information has been appropriately anonymized to maintain the confidentiality and privacy of the subjects.

## Supporting information

Additional File 1 — Supporting Information

Additional File 2 — Supplementary Tables.
**TABLE S1** Histopathological subtypes of breast cancer identified in *MMTV‐Ncaph* transgenic mice.
**TABLE S2** (A) Clinical characteristics of a cohort of patients with luminal A tumours, with good and poor outcome after 10 years of follow‐up at the University Hospital of Salamanca. (B) Intratumoural levels of NCAPH, determined by immunohistochemistry (IHC) and evaluation of associations with different tumour characteristics.
**TABLE S3** List of 64 genes associated with high levels of *NCAPH*.
**TABLE S4** Gene Ontology (GO) identifies a list of biological functions in which the coregulated Ncaph genes participate.
**TABLE S5** Univariate analysis using least absolute shrinkage and selection operator (LASSO) was used to identify associations between the levels of transcripts associated with *Ncaph* in the BX‐*Neu^+^* cohort and survival.
**TABLE S6** Association of genes correlated with *Ncaph* in mouse tumours with outcome in different subtypes of breast cancer defined by PAM50 and luminal B HER2‐positive tumours, defined by receptor expression.
**TABLE S7** Univariate analysis using Cox regression to identify associations between the transcript levels associated with *NCAPH* and relapse‐free survival (RFS) in the human Gene Expression‐based Outcome for Breast Cancer Online (GOBO) cohort.
**TABLE S8**. List of genes whose transcripts are components of the Gene Signature for Luminal A 10 (GSLA10).

**Additional File 3** — **Supplementary Figures**.
**FIGURE S1**. NCAPH and breast cancer prognosis: a preliminary analysis.
**FIGURE S2**. Effect of NCAPH induction on breast cancer cells of basal origin.
**FIGURE S3**. Comparative analysis of pERBB2 and ERBB2 levels in MMTV‐*ErbB2* and MMTV‐*Ncaph^ErbB2^* mouse tumours and quantification of Caspase 3.
**FIGURE S4**. Analysis of *Ncaph* levels in local tumour growth response and metastatic potential post‐chemotherapy.
**FIGURE S5**. The gene signature is correlated with intratumoural *Ncaph* levels in the BX‐*Neu^+^* mouse cohort.
**FIGURE S6**. The least absolute shrinkage and selection operator (LASSO) regression model is used to predict prognosis in luminal A breast cancer.

## Data Availability

All data generated or analysed during this study are included in this published article (and its supporting information files). The datasets generated and/or analysed during the current study are available from the corresponding authors upon reasonable request. The datasets analysed during the current study are available from GOBO (http://co.bmc.lu.se/gobo),^41^ METABRIC (https://www.cbioportal.org/) and TCGA‐BRCA (https://www.cbioportal.org/).
