# Supplementary material for: NCAPH drives breast cancer progression and identifies a gene signature that predicts luminal a tumour recurrence
Source: Clin Transl Med. 2024 Feb 12;14(2):e1554. doi: 10.1002/ctm2.1554 (PMC10859882; doi:10.1002/ctm2.1554)
Supplement: Supplementary file 1 — Additional File 1 — Supporting Information [file CTM2-14-e1554-s003.docx]

**Additional File 1: Supplemental Methods**

**PCR screening**

The presence of the *ErbB2* and *Ncaph* transgenes was detected in tail DNA by PCR performed in a total volume of 20 μl in 200 μl PCR tubes with the following reagents:10× PCR buffer (2 μl) with MgCl_2_ (1 mM; Takara, Otsu, Shiga, Japan), dNTPs (200 μM; Takara), oligonucleotides (5 μM), 10 units of Taq polymerase (Takara), ddH_2_O, and DNA (1 μl, approximately 200 ng). For *Ncaph* amplification, The oligonucleotides used were 5′-TTCTGGCTGGCGTGGAAATA-3′ and 5′-GGCCAATAAGCGAGGTGAGT-3′. The PCR program consisted of one cycle of 5 min at 94 °C; 12 cycles of 20 s at 94 °C and 30 s at 62 °C (this temperature was decreased by 0.5 °C in each cycle), and 35 s at 72 °C; 30 cycles of 20 s at 94 °C, 30 s at 58 °C, and 35 s at 72 °C, with a final extension phase of 2 min at 72 °C. For *ErbB2* amplification, the oligonucleotides used were 5′-CAGGTGCAAGCACTATTGACC-3′ and 5′-CTCAGAGCTCAGATCAGAACC-3,’ and the PCR program consisted of one cycle of 3 min at 94 °C; 12 cycles of 20 s at 94 °C, 30 s at 64 °C (this temperature was decreased by 0.5 °C in each cycle), and 35 s at 72 °C; 25 cycles of 20 s at 94 °C, 30 s at 58 °C, and 35 s at 72 °C; and finally, a 2 min extension at 72 °C. All PCR products were analyzed on 1% agarose gels.

**Evaluation of tumor pathophenotypes**

Female transgenic mice were palpated once weekly to check for primary mammary tumors and differentiate the temporal stages of tumor progression and outcome. In the first group, we distinguished (i) tumor latency, defined as the time between the date of birth and the age at which the first mammary tumor was detected; (ii) disease duration, defined as the period between the appearance of the first breast tumor and the time of death; and (iii) lifespan, defined as the sum of tumor latency and disease duration.

With regard to tumor progression, we first differentiated the number of breast tumors by counting all tumors visible at necropsy. We distinguished between the absolute tumor number, incidence, and multiplicity. Tumor incidence was defined as the proportion of female mice that generated at least one mammary tumor during the experiment. Tumor multiplicity was defined as the proportion of female mice that developed two or more tumors after a given time. We also compared local tumor progression in mice once an initial tumor appeared and measured the tumor volume every week. The tumor volume was measured weekly using a digital caliper, with dimensions recorded in millimeters. The volume calculation employed the established formula: (greater diameter × lesser diameter²) / 2. The growth velocity was determined using the formula: (final volume - initial volume) / duration of the illness in weeks, yielding units of cubic millimeters per week. We also considered the final tumor weight and volume at the time of necropsy. In addition, we examined distant tumor progression by quantifying the incidence and multiplicity of lung metastases, as tumors from MMTV-*ErbB2* mice only disseminate to the lung[^60^](#_ENREF_60). We also considered the absolute number, incidence, and multiplicity of metastases. We defined the incidence of metastasis as the proportion of female mice with at least one mammary tumor metastasis in the lung at the time of necropsy. The absolute number of metastases was the extreme value of the metastatic distribution range for each genotype. Metastasis multiplicity was defined as the proportion of female mice that developed more than one lung metastasis during the experiment. Mice were sacrificed when they showed signs of sickness, when a rapidly growing tumor had developed, or when wounds were observed.

To assess tumor response to chemotherapy, we evaluated the tumor growth rate at various disease stages: pre-treatment, during treatment, and post-treatment. Growth rate slopes were determined by plotting the logarithm of the tumor volume on the Y-axis against time on the X-axis, with units in inverse weeks (weeks^-1). Besides the tumor growth rate (GR), we introduced two variables to quantify chemotherapy-induced changes in tumor growth dynamics:

(i) Response During Treatment (RDT): This is calculated as the difference in GR before and during chemotherapy.

(ii) Evolution Changes Induced by Chemotherapy (ECIC): This is calculated as the difference in GR before and after chemotherapy.

A higher value of RDT and ECIC indicates a more effective treatment response or outcome.

**Cell viability**

Two 10 cm dishes were seeded with 1 x10^6^ MCF-7 or BT549-NCAPH inducible cells per dish, and vehicle alone or doxycycline (10 µg/ml) was added. The cells were grown for 17 days, and after checking for NCAPH induction, 35 x10^3^ cells were seeded into three 12-well plates and fed fresh medium. The plates were fixed for 24, 48, and 72 h with 4% PFA at room temperature and stored in PBS with 0.05% azide at 4 °C before staining with crystal violet for 30 min at room temperature. The plates were washed with tap water, crystal violet was removed from the cells with 1% Triton X-100 in PBS, and the OD was measured at 570 nm. To assess cell viability in response to treatment with tamoxifen (1 μM), docetaxel (0.5 μM), or doxorubicin (0.5 μM) for different times, samples were processed similarly as above. Viability assays were performed at least three times for each drug, and the ratio of surviving cells to controls was determined. Cell viability was determined in MTT assays following the manufacturer’s instructions (Sigma-Aldrich, St.Louis, Missouri, USA #M5655).

**Soft agar assay**

MCF-7-NCAPH inducible cells were induced with doxycycline (10 µg/ml) for 14 days before the soft agar colony formation assays were performed. Briefly, 5 x10^3^ cancer cells were triple seeded in 1.5 mL of tissue culture medium with 1% glutamine and antibiotics, and 0.4% soft agar was layered onto 0.8% solidified agar in tissue culture medium in 6-well plates. After incubation for 14 days, the colony foci were stained with 0.005% crystal violet, and those larger than 50 µm were counted under a dissecting microscope. The experiments were carried out in triplicate.

**Quantitative real-time PCR (qPCR)**

Total RNA was isolated from the cells using the RNAeasy Mini kit (QIAGEN) according to the manufacturer's instructions and quantified using a NanoDrop® spectrophotometer. RNA (3 μg) was reverse-transcribed in a final volume of 20 μL using an NZY First-Strand cDNA Synthesis Kit (NZYTech), according to the manufacturer's instructions. To quantify *NCAPH* and *GAPDH* gene expression, SYBR Green reagent was used in the PCR reaction mixture (10 μL final volume). The primers used were: *NCAPH* F-GCCATAGGGCAGAGACTGAG and *NCAPH* R-GTTCCCAACAGGTCCCACAA; *GAPDH* F-CTGCACCACCAACTGCTTAG and R-GTCTTCTGGGTGGCAGTGAT. PCR was performed using a Fast Real-Time PCR System (Applied Biosystems), and the transcript levels were normalized relative to those of GAPDH, which was used as an endogenous control. Gene expression was analyzed in triplicate and quantified using the ^ΔΔ^Ct method.

**Genomic instability analysis**

Two 10 cm dishes were seeded with 1x10^6^ MCF-7-NCAPH inducible cells per dish, and vehicle alone or doxycycline (10 µg/ml) was added to the dishes. The cells were grown for 17 days, and after checking for NCAPH induction, 1x10^6^ cells were seeded in 10 cm dishes. To achieve cell synchronization, FBS was removed from the medium 24 h prior to adding the newly completed medium. The next day, the cells were treated with 30 mM H_2_O_2_ in serum-free medium for 30 min on ice. The medium was then changed by completion, plates were placed in the incubator for the time required according to the experiment, and γH2AX and pCHEK1 were monitored as markers of Double-Stranded Breaks (DSBs). For chromosomal aberrations, cells were seeded on coverslips for 24 h in the presence or absence of doxycycline, and the coverslips were fixed with 4% PFA and mounted on glass slides with DAPI using the Gold Antifade reagent (Invitrogen; #P36935). Well-spread metaphases were identified with a 100x objective using a Zeiss Axioplan 2 microscope. Metaphase spreads were identified and scored by eye for micronucleus formation and lagging chromosome (CIN).

**Immunofluorescence microscopy**

Cells were plated and grown on glass coverslips for 48 h, fixed for 10 min with 4% PFA at room temperature, and then stored in PBS with 0.05% sodium azide at 4 °C until immunostaining. The cells were permeabilized by incubating for 3 min at 4 °C in PBS containing 0.5% Triton X-100, and the coverslips were then incubated overnight at 4 °C with a 1:200 dilution of the primary antibody against Ki67 (Thermo Fisher Scientific, #PA1-21520). After rinsing thoroughly, the coverslips were incubated with Alexa Fluor 488 or 594 goat anti-mouse-IgG or anti-rabbit-IgG secondary antibodies (Invitrogen) and then mounted on glass slides with DAPI using Gold antifade reagent (#P36935: Invitrogen). Immunofluorescence was analyzed using a Zeiss microscope (Zeiss Axioplan 2).

To calculate the proliferation ratio using the Ki67 marker, we counted the number of positively stained tumor cells in each image/field and the number of tumor cells in each image. The percentage of Ki67 positive cells was calculated as the number of positive tumor cells/number of all tumor cells ×100.

**Analysis and quantification of NCAPH immunohistochemistry**

For the analysis and quantification of NCAPH immunohistochemistry, we utilized Fiji, an open-source image processing package derived from ImageJ2. The process involved manually measuring the average staining intensity.

Our detailed methodology is as follows: We converted images to RGB format. Subsequently, we executed a color deconvolution using the H-DAB (hematoxylin-diaminobenzidine) vectors. This process yielded three images, of which the one associated with DAB staining (Colour 2) was used for quantification. Our region of interest (ROI) was selected, followed by measurement and analysis of results. Fiji provided an intensity value, termed 'Mean Intensity', measured in arbitrary units as described by Nguyen DH *et al.* (2013). These values ranged from 0 (maximum intensity) to 250 (minimum intensity). To interpret these values effectively, we calculated the Reciprocal Intensity (RI) as per the method of Nguyen DH *et al. (*2013), where higher RI values indicate greater staining intensity. Unlike Nguyen DH *et al.* (2013), we adjusted for the background intensity of each image, using this value instead of the default 250.

The formulas applied were as follows:

Reciprocal Intensity (RI) = Mean Intensity (background) - Mean Intensity (epithelium).

Normalized RI = RI / Mean Intensity (background).
