# Supplementary material for: NCAPH drives breast cancer progression and identifies a gene signature that predicts luminal a tumour recurrence
Source: Clin Transl Med. 2024 Feb 12;14(2):e1554. doi: 10.1002/ctm2.1554 (PMC10859882; doi:10.1002/ctm2.1554)
Supplement: Supplementary file 2 — Additional File 2 — Supplementary Tables. TABLE S1 Histopathological subtypes of breast cancer identified in MMTV‐Ncaph transgenic mice. TABLE S2 (A) Clinical characteristics of a cohort of patients with luminal A tumours, with good and poor outcome after 10 years of follow‐up at the University Hospital of Salamanca. (B) Intratumoural levels of NCAPH, determined by immunohistochemistry (IHC) and evaluation of associations with different tumour characteristics. TABLE S3 List of 64 genes associated with high levels of NCAPH. TABLE S4 Gene Ontology (GO) identifies a list of biological functions in which the coregulated Ncaph genes participate. TABLE S5 Univariate analysis using least absolute shrinkage and selection operator (LASSO) was used to identify associations between the levels of transcripts associated with Ncaph in the BX‐Neu+ cohort and survival. TABLE S6 Association of genes correlated with Ncaph in mouse tumours with outcome in different subtypes of breast cancer defined by PAM50 and luminal B HER2‐positive tumours, defined by receptor expression. TABLE S7 Univariate analysis using Cox regression to identify associations between the transcript levels associated with NCAPH and relapse‐free survival (RFS) in the human Gene Expression‐based Outcome for Breast Cancer Online (GOBO) cohort. TABLE S8. List of genes whose transcripts are components of the Gene Signature for Luminal A 10 (GSLA10). [file CTM2-14-e1554-s002.docx]

# Additional File 2: Supplementary Tables

Table S1 page 2

Table S2 page 3

Table S3 page 4

Table S4… page 5

Table S5… page 6

Table S6… page 7

Table S7………………..page 8

Table S8. Page 9

1

**Table S1. Histopathological subtypes of breast cancer identified in the *MMTV-Ncaph* transgenic mice**.

| **Mouse Line** | | **Nulliparous/ Parous** | **Mouse ID** | **Tumor type** | **Histopathology** |
| --- | --- | --- | --- | --- | --- |
| *Ncaph* #1 | First cohort | nulliparous | **T131** | Breast Tumor | Sarcomatoid carcinoma |
|  |  |  | **T164** | Breast Tumor | Poorly differentiated invasive ductal carcinoma |
|  |  |  | **T182** | Breast Tumor | Metaplastic carcinoma with squamous differentiation |
|  |  | parous | **T303** | Breast Tumor-1 | Poorly differentiated invasive ductal carcinoma |
|  |  |  |  | Breast Tumor-2 | Well-differentiated invasive ductal carcinoma |
|  |  |  |  | Breast Tumor-3 | Poorly differentiated invasive ductal carcinoma |
|  |  |  | **T346** | Breast Tumor | Invasive ductal carcinoma with mixoid differentiation |
|  | Second cohort | parous | **T671** | Breast Tumor | Metaplastic carcinoma with squamous differentiation |
|  |  |  | **T711** | Breast Tumor | Well-differentiated invasive ductal carcinoma with papillary differentation |
|  |  |  | **T984** | Breast Tumor | Well-differentiated invasive ductal carcinoma |
|  |  |  | **T1163** | Breast Tumor-1 | Metaplastic carcinoma with squamous differentiation |
|  |  |  |  | Breast Tumor-2 | Metaplastic carcinoma with squamous differentiation |
| *Ncaph* #2 | | parous | **T858** | Breast Tumor | Well-differentiated invasive ductal carcinoma with papillary differentation |
|  |  |  | **T912** | Breast Tumor | Poorly differentiated invasive ductal carcinoma |
|  |  |  | **T918** | Breast Tumor | Sarcomatoid carcinoma |
|  |  |  | **T937** | Breast Tumor | Well-differentiated invasive ductal carcinoma with papillary differentation |
|  |  |  | **T1035** | Breast Tumor | Sarcomatoid carcinoma |
|  |  |  | **T1084** | Breast Tumor | Sarcomatoid carcinoma |

2

**Table S2.**

1. **Clinical characteristics of a cohort of patients with luminal A tumors, with good and poor evolution after 10 years of follow-up at the University Hospital of Salamanca.** IDC, infiltrating ductal carcinoma; MC, mucinous carcinoma; CoC, colloid carcinoma; ILC, infiltrating lobular carcinoma; MPC, micropapillary carcinoma.

| **Patient ID** | **Age** | **Tumor grade** | **TNM** | **Tumor stage** | **Tumor size (mm)** | **Diagnosis** | **ER%** | **PR%** | **Ki67%** | **Evolution** | **Liver Metastasis after 5 years Follow-up** |
| --- | --- | --- | --- | --- | --- | --- | --- | --- | --- | --- | --- |
| 1 | 58 | 1 | pT2N1 | IIB | - | IDC | 90 | 90 | 25 | Poor | Yes |
| 2 | 70 | 1 | pT1cN0 | IA | 20 | MC | 90 | 90 | 5 | Poor | Yes |
| 3 | 65 | 2 | pT1cN0 | IA | 10 | IDC | 90 | 80 | 15 | Poor | Yes |
| 4 | 62 | 3 | cT1cN1 | IIA | 6 | IDC | 90 | - | 5 | Poor | Yes |
| 5 | 45 | 2 | pT1cN1 | IIA | 150 | IDC + CoC | 90 | 70 | 20 | Poor | Yes |
| 6 | 56 | - | cT4bN1M1 | IV | 30 | IDC | 90 | 90 | 15 | Poor | Yes |
| 7 | 41 | 3 | pT2N1 | IIB | 45 | IDC | 90 | 30 | 15 | Poor | Yes |
| 8 | 37 | 2 | Metastatic | IV | - | IDC | 80 | 30 | 10 | Poor | Yes |
| 9 | 69 | 2 | pT1cN0M0 | IA | 13 | IDC | 100 | 60 | 10 | Good | No |
| 10 | 67 | 1 | pT1cN0M0 | IA | 16 | IDC | 70 | 50 | 10 | Good | No |
| 11 | 64 | 2 | pT1bN1M0 | IIA | 8 | IDC | 100 | 30 | 3 | Good | No |
| 12 | 61 | 1 | pT1aN0M0 | IA | 4 | IDC | 70 | 50 | 2 | Good | No |
| 13 | 60 | 2 | pT1bN0M0 | IA | 6 | IDC | 70 | 50 | 6 | Good | No |
| 14 | 47 | 2 | pT1cN0M0 | IA | 20 | IDC | 70 | 90 | 6 | Good | No |
| 15 | 86 | 2 | pT1cN0M0 | IA | 12 | IDC | 90 | 90 | 10 | Good | No |
| 16 | 62 | 1 | pT1aN0M0 | IA | 7 | IDC | 100 | 60 | 5 | Good | No |
| 17 | 63 | 2 | pT1cN0M0 | IA | 20 | IDC | 99 | 50 | 6 | Good | No |
| 18 | 47 | 1 | pT1cN0M0 | IA | 15 | IDC | 80 | 99 | 6 | Good | No |
| 19 | 69 | - | pT2N2M0 | IIIA | 27 | ILC | 100 | 100 | 2 | Good | No |
| 20 | 64 | 2 | pT1bN0M0 | IA | 8 | MPC + IDC | 95 | 100 | 10 | Good | No |
| 21 | 43 | 1 | pTbN0M0 | IA | - | - | 90 | 100 | 5 | Good | No |
| 22 | 76 | - | pT2N1M0 | IIB | 50 | MC | 100 | 100 | 5 | Good | No |
| 23 | 61 | 2 | pT2N1M0 | IIB | 30 | IDC | 95 | 85 | 10 | Good | No |
| 24 | 53 | 1 | pT1bN0M0 | IA | 8 | IDC | 90 | 90 | 5 | Good | No |
| 25 | 60 | 2 | pT1bN0M0 | IA | 6 | IDC | 80 | 30 | 5 | Good | No |
| 26 | 47 | - | pT1cN0M0 | IA | 18 | ILC | 75 | 50 | 5 | Good | No |
| 27 | 84 | 2 | pT1cN2M0 | IIIA | 15 | IDC | 75 | 75 | 5 | Good | No |
| 28 | 53 | 2 | pTisN0M0 | 0 | - | - | +++ | +++ | - | Good | No |

1. **Intratumoral levels of NCAPH, determined by immunohistochemistry and the evaluation of associations with different tumor characteristics.** The intensity of NCAPH staining in the tumor epithelium was quantified using the Fiji software (Schindelin *et al.,* 2012). For the evaluation, the reciprocal intensity that, in each case, was normalized by the background of the image was calculated (Nguyen *et al.,* 2013); see the Materials and Methods section. Median, interquartile range.

| **Evolution** | **Good** | | | **Poor** | | | | ***P*** | **Test** |
| --- | --- | --- | --- | --- | --- | --- | --- | --- | --- |
|  | N = 20 | | | N = 8 | | | |  |  |
|  | 25.06 (18.94 - 30.60) | | | 43.06 (24.74 - 56.44) | | | | 0.0157* | *Mann–Whitney U* |
|  |  |  |  |  |  |  |  |  |  |
| **Tumor grade** | **1** | | **2** | | | **3** | | ***P*** | **Test** |
|  | N = 8 | | N = 14 | | | N = 2 | |  |  |
|  | 29.43 (21.65 - 41.17) | | 24.15 (16.50 - 33.98) | | | 46.54 (34.49 - 58.59) | | 0.1393 | *Kruskal–Wallis* |
|  |  |  |  |  |  |  |  |  |  |
| **Tumor stage** | **0** | **IA** | **IIA** | | **IIB** | **IIIA** | **IV** | ***P*** | **Test** |
|  | N = 1 | N = 16 | N = 3 | | N = 4 | N = 2 | N = 2 |  |  |
|  | 30.4 | 29.43 (17.21 - 34.00) | 23.01 (21.49 - 34.49) | | 35.99 (21.41 - 54.83) | 24.53 (23.77 - 25.30) | 33.62 (14.74 - 52.49) | 0.9293 | *Kruskal–Wallis* |
|  |  |  |  |  |  |  |  |  |  |
| **Ki67 (≥15)** | **Low (< 15)** | | | | **High (≥ 15)** | | | ***P*** | **Test** |
|  | N = 22 | | | | N = 5 | | |  |  |
|  | 26.88 (19.02 - 33.75) | | | | 42.59 (18.12 - 51.06) | | | 0.3769 | *Mann–Whitney U* |
|  |  |  |  |  |  |  |  |  |  |
|  |  |  |  |  |  |  |  |  |  |
| **N** | **Early stage (0, IA)** | | | | **Late stage (IIA, IIB, IIIA, IV)** | | | ***P*** | **Test** |
|  | N = 17 | | | | N = 11 | | |  |  |
|  | 29.86 (17.77 - 33.88) | | | | 25.3 (21.49 - 43.53) | | | 0.7461 | *Mann–Whitney U* |
|  |  |  |  |  |  |  |  |  |  |

**Table S3. List of 64 genes associated with high levels of *NCAPH.*** The selection criteria were a < - 2-fold change for underexpressed genes and a > 2-fold change for overexpressed genes, with a value of *p* < 0.05.

| **Gene symbol** | **Fold change** | ***p*** | **FDR *p*** |
| --- | --- | --- | --- |
| *Dpp10* | 4.91 | 1.20E-09 | 1.34E-07 |
| *4930503L19Rik* | 3.88 | 4.67E-05 | 0.001 |
| *Tspan1* | 3.56 | 1.37E-11 | 2.74E-09 |
| *Gpx2* | 3.32 | 0.0005 | 0.0057 |
| *Slc38a1* | 3.1 | 7.98E-06 | 0.0002 |
| *Slc38a1* | 2.99 | 3.29E-06 | 0.0001 |
| *Unc79* | 2.95 | 8.81E-06 | 0.0003 |
| *Gjb1* | 2.66 | 1.09E-11 | 2.26E-09 |
| *Dcpp2* | 2.65 | 0.0056 | 0.0346 |
| *Cpe* | 2.58 | 0.0213 | 0.0802 |
| *Dcpp1; Dcpp2* | 2.53 | 0.0078 | 0.0434 |
| *Bub1* | 2.38 | 3.98E-14 | 1.83E-11 |
| *Rrm2* | 2.38 | 5.70E-12 | 1.31E-09 |
| *Shc4* | 2.31 | 7.43E-06 | 0.0002 |
| *H2afy2* | 2.28 | 4.53E-11 | 7.72E-09 |
| *Pde1c* | 2.26 | 1.05E-07 | 6.28E-06 |
| *Gm27343* | 2.26 | 1.03E-05 | 0.0003 |
| *Pgf* | 2.24 | 0.0002 | 0.0024 |
| *Pglyrp1* | 2.21 | 9.86E-05 | 0.0017 |
| *Slc35f1* | 2.18 | 6.76E-05 | 0.0013 |
| *Prom1* | 2.17 | 1.03E-05 | 0.0003 |
| *Psrc1* | 2.16 | 2.71E-11 | 5.12E-09 |
| *Bcar3* | 2.16 | 0.0002 | 0.0034 |
| *St8sia6* | 2.14 | 2.30E-05 | 0.0005 |
| *Oip5* | 2.13 | 2.92E-21 | 2.11E-17 |
| *Krt23* | 2.12 | 4.30E-10 | 5.25E-08 |
| *Ncaph* | 2.11 | 1.39E-21 | 1.34E-17 |
| *4631405J19Rik* | 2.1 | 2.91E-10 | 3.76E-08 |
| *Gbp3* | 2.1 | 0.0004 | 0.0051 |
| *Apol9a* | 2.09 | 0.0022 | 0.018 |
| *Cd177* | 2.09 | 0.015 | 0.0649 |
| *Bub1b* | 2.07 | 8.09E-17 | 9.75E-14 |
| *Apol9b* | 2.07 | 0.0017 | 0.0151 |
| *Rad51* | 2.04 | 1.65E-17 | 2.52E-14 |
| *Ifi27l2a* | 2.04 | 0.0003 | 0.0036 |
| *Cdc25c* | 2.03 | 7.59E-14 | 3.33E-11 |
| *Higd1a* | 2.03 | 1.48E-07 | 8.45E-06 |
| *Casc5* | 2.02 | 2.45E-14 | 1.20E-11 |
| *Pole2* | 2.02 | 1.75E-13 | 6.65E-11 |
| *Mfsd4* | 2.02 | 7.56E-06 | 0.0002 |
| *Wap* | -2.02 | 0.0001 | 0.002 |
| *Wnt5a* | -2.03 | 3.80E-06 | 0.0001 |
| *Muc15* | -2.03 | 0.0014 | 0.0131 |
| *Angpt1* | -2.06 | 6.05E-06 | 0.0002 |
| *Gm20946* | -2.08 | 1.06E-07 | 6.32E-06 |
| *Gm7697* | -2.08 | 4.01E-07 | 1.99E-05 |
| *Serpinb5* | -2.11 | 8.52E-05 | 0.0015 |
| *Cytip* | -2.12 | 2.76E-05 | 0.0006 |
| *Crispld2* | -2.13 | 0.001 | 0.0103 |
| *Gm9495* | -2.14 | 2.78E-07 | 1.45E-05 |
| *4930467E23Rik* | -2.14 | 1.13E-06 | 4.75E-05 |
| *Pik3c2g* | -2.23 | 3.79E-05 | 0.0008 |
| *Spp1* | -2.4 | 5.84E-05 | 0.0011 |
| *Scrg1* | -2.43 | 0.0001 | 0.002 |
| *Slc38a3* | -2.47 | 2.07E-05 | 0.0005 |
| *Btn1a1* | -2.56 | 0.014 | 0.0622 |
| *Rdh12* | -2.58 | 0.044 | 0.1202 |
| *Sftpd* | -2.65 | 7.71E-06 | 0.0002 |
| *Csn1s2a* | -2.89 | 0.0044 | 0.0293 |
| *Csn1s1* | -2.94 | 0.0252 | 0.088 |
| *Slc6a15* | -3.08 | 0.0001 | 0.002 |
| *Spink5* | -3.31 | 1.23E-07 | 7.18E-06 |
| *Csn2* | -3.62 | 0.0027 | 0.0205 |
| *Lao1* | -3.94 | 0.0004 | 0.0048 |

**Table S4.** Gene Ontology identifies a list of biological functions in which the co-regulated Ncaph genes participate.

| **Ontology** | **ID** | **Description** | **Gene ratio** | **Bg ratio** | ***p*** | **Adjusted *p*** | ***q*** | **Gene ID** | **Frequency** |
| --- | --- | --- | --- | --- | --- | --- | --- | --- | --- |
| BP | GO:1903046 | meiotic cell cycle process | 6/60 | 198/23210 | 1.21E-05 | 0.016241577 | 0.013207352 | *Ncaph/Rad51/Bub1b/Bub1/Cdc25c/Wnt5a* | 6 |
| BP | GO:0070192 | chromosome organization involved in the meiotic cell cycle | 4/60 | 71/23210 | 3.44E-05 | 0.022937791 | 0.01865259 | *Ncaph/Rad51/Bub1b/Bub1* | 4 |
| BP | GO:0140013 | meiotic nuclear division | 5/60 | 179/23210 | 9.99E-05 | 0.03374637 | 0.027441927 | *Ncaph/Rad51/Bub1b/Bub1/Wnt5a* | 5 |
| BP | GO:0015804 | neutral amino acid transport | 3/60 | 35/23210 | 0.00010134 | 0.03374637 | 0.027441927 | *Slc38a1/Slc38a3/Slc6a15* | 3 |
| BP | GO:0051321 | meiotic cell cycle | 6/60 | 314/23210 | 0.000158354 | 0.035765934 | 0.0290842 | *Ncaph/Rad51/Bub1b/Bub1/Cdc25c/Wnt5a* | 6 |
| BP | GO:0007059 | chromosome segregation | 6/60 | 315/23210 | 0.000161108 | 0.035765934 | 0.0290842 | *Ncaph/Oip5/Bub1b/Knl1/Bub1/Psrc1* | 6 |
| BP | GO:0050918 | positive chemotaxis | 3/60 | 44/23210 | 0.000201701 | 0.03838089 | 0.031210633 | *Wnt5a/Angpt1/Pgf* | 3 |
| BP | GO:0045144 | meiotic sister chromatid segregation | 2/60 | 10/23210 | 0.000291806 | 0.043187303 | 0.03511912 | *Bub1b/Bub1* | 2 |
| BP | GO:0051177 | meiotic sister chromatid cohesion | 2/60 | 10/23210 | 0.000291806 | 0.043187303 | 0.03511912 | *Bub1b/Bub1* | 2 |
| BP | GO:0070601 | centromeric sister chromatid cohesion | 2/60 | 11/23210 | 0.000356059 | 0.047348103 | 0.038502606 | *Bub1b/Bub1* | 2 |
| BP | GO:0007135 | meiosis II | 2/60 | 12/23210 | 0.000426559 | 0.047348103 | 0.038502606 | *Bub1b/Bub1* | 2 |
| BP | GO:0061983 | meiosis II cell cycle process | 2/60 | 12/23210 | 0.000426559 | 0.047348103 | 0.038502606 | *Bub1b/Bub1* | 2 |
| BP | GO:0098813 | nuclear chromosome segregation | 5/60 | 256/23210 | 0.000522804 | 0.050347649 | 0.040941782 | *Ncaph/Bub1b/Knl1/Bub1/Psrc1* | 5 |
| BP | GO:0000070 | mitotic sister chromatid segregation | 4/60 | 144/23210 | 0.000529179 | 0.050347649 | 0.040941782 | *Ncaph/Bub1b/Bub1/Psrc1* | 4 |
| BP | GO:0000280 | nuclear division | 6/60 | 408/23210 | 0.000639085 | 0.056750729 | 0.046148649 | *Ncaph/Rad51/Bub1b/Bub1/Psrc1/Wnt5a* | 6 |
| BP | GO:0060688 | regulation of morphogenesis of a branching structure | 3/60 | 67/23210 | 0.000699423 | 0.05701089 | 0.046360207 | *Wnt5a/Pgf/Cpe* | 3 |
| BP | GO:0050777 | negative regulation of immune response | 4/60 | 158/23210 | 0.000749452 | 0.05701089 | 0.046360207 | *Spink5/Angpt1/Pglyrp1/Gpx2* | 4 |
| BP | GO:0002739 | regulation of cytokine secretion involved in immune response | 2/60 | 16/23210 | 0.000770417 | 0.05701089 | 0.046360207 | *Wnt5a/Angpt1* | 2 |
| BP | GO:0044406 | adhesion of symbiont to host | 2/60 | 17/23210 | 0.000871689 | 0.061109961 | 0.049693496 | *Sftpd/Gbp3* | 2 |
| CC | GO:0000777 | condensed chromosome kinetochore | 3/61 | 35/23436 | 0.000103481 | 0.011175974 | 0.008932065 | *Bub1b/Knl1/Bub1* | 3 |
| CC | GO:0000793 | condensed chromosome | 5/61 | 209/23436 | 0.000213206 | 0.011507335 | 0.009196896 | *Ncaph/Rad51/Bub1b/Knl1/Bub1* | 5 |
| CC | GO:0000779 | condensed chromosome, centromeric region | 3/61 | 51/23436 | 0.000319648 | 0.011507335 | 0.009196896 | *Bub1b/Knl1/Bub1* | 3 |
| CC | GO:0000940 | condensed chromosome outer kinetochore | 2/61 | 15/23436 | 0.000684617 | 0.014059969 | 0.011237012 | *Bub1b/Bub1* | 2 |
| CC | GO:0000794 | condensed nuclear chromosome | 4/61 | 155/23436 | 0.000716525 | 0.014059969 | 0.011237012 | *Ncaph/Rad51/Bub1b/Bub1* | 4 |
| CC | GO:0000778 | condensed nuclear chromosome kinetochore | 2/61 | 16/23436 | 0.000781109 | 0.014059969 | 0.011237012 | *Bub1b/Bub1* | 2 |
| CC | GO:0098687 | chromosomal region | 5/61 | 297/23436 | 0.001052817 | 0.016243459 | 0.012982101 | *Oip5/Rad51/Bub1b/Knl1/Bub1* | 5 |
| CC | GO:0000775 | chromosome, centromeric region | 4/61 | 190/23436 | 0.001522989 | 0.020560345 | 0.016432245 | *Oip5/Bub1b/Knl1/Bub1* | 4 |
| CC | GO:0000780 | condensed nuclear chromosome, centromeric region | 2/61 | 29/23436 | 0.002585868 | 0.031030414 | 0.024800136 | *Bub1b/Bub1* | 2 |
| CC | GO:0000776 | kinetochore | 3/61 | 132/23436 | 0.004953334 | 0.053496004 | 0.042755091 | *Bub1b/Knl1/Bub1* | 3 |
| MF | GO:0005172 | vascular endothelial growth factor receptor binding | 2/57 | 15/22707 | 0.000636555 | 0.062583804 | 0.047241237 | *Angpt1/Pgf* | 2 |
| MF | GO:0015171 | amino acid transmembrane transporter activity | 3/57 | 73/22707 | 0.000823471 | 0.062583804 | 0.047241237 | *Slc38a1/Slc38a3/Slc6a15* | 3 |

**Table S5.** Bivariant analyses using Cox regression were used to identify associations between the levels of transcripts associated with Ncaph in the BX-*Neu+* cohort and survival. Transcripts with a P value lower than 0.25 were chosen to construct the multivariate model using LASSO regression.

|  | **Cox regression** | | | |
| --- | --- | --- | --- | --- |
|  | **Beta** | **HR (95% CI for HR)** | **Wald test** | **p.value** |
| ***Ncaph*** | 0.25 | 1.3 (0.86-1.9) | 1.6 | 0.21 |
| ***Oip5*** | 0.89 | 2.4 (1.6-3.7) | 18 | 2.20E-05 |
| ***Rad51*** | 0.75 | 2.1 (1.5-3.1) | 15 | 9.10E-05 |
| ***Bub1b*** | 0.59 | 1.8 (1.3-2.6) | 10 | 0.0012 |
| *Casc5* | 0.18 | 1.2 (0.79-1.8) | 0.72 | 0.4 |
| ***Bub1*** | 0.59 | 1.8 (1.3-2.5) | 13 | 0.00034 |
| ***Cdc25c*** | 0.76 | 2.1 (1.4-3.2) | 13 | 0.00024 |
| ***Pole2*** | 0.51 | 1.7 (1.2-2.2) | 12 | 0.00063 |
| ***Rrm2*** | 0.28 | 1.3 (1-1.7) | 3.8 | 0.051 |
| ***Gjb1*** | 0.51 | 1.7 (1.3-2.2) | 12 | 0.00041 |
| ***Tspan1*** | 0.097 | 1.1 (0.93-1.3) | 1.3 | 0.25 |
| ***Psrc1*** | 0.23 | 1.3 (0.96-1.7) | 2.9 | 0.09 |
| ***H2afy2*** | 0.55 | 1.7 (1.3-2.2) | 18 | 2.80E-05 |
| ***Krt23*** | 0.35 | 1.4 (1.1-1.9) | 6.1 | 0.014 |
| *Spink5* | -0.097 | 0.91 (0.73-1.1) | 0.81 | 0.37 |
| ***Higd1a*** | 0.52 | 1.7 (1.3-2.1) | 19 | 1.40E-05 |
| ***Slc38a1*** | 0.15 | 1.2 (1-1.3) | 3.9 | 0.049 |
| *Wnt5a* | 0.069 | 1.1 (0.87-1.3) | 0.4 | 0.53 |
| *Angpt1* | 0.043 | 1 (0.87-1.3) | 0.22 | 0.64 |
| *Sftpd* | 0.041 | 1 (0.88-1.2) | 0.21 | 0.64 |
| ***Prom1*** | 0.29 | 1.3 (1.1-1.7) | 6 | 0.014 |
| *Slc38a3* | 0.021 | 1 (0.83-1.3) | 0.04 | 0.84 |
| *Pik3c2g* | -0.051 | 0.95 (0.74-1.2) | 0.16 | 0.69 |
| *Spp1* | 0.002 | 1 (0.88-1.1) | 0 | 0.98 |
| *Serpinb5* | 0.062 | 1.1 (0.89-1.3) | 0.44 | 0.51 |
| *Pglyrp1* | -0.08 | 0.92 (0.79-1.1) | 0.99 | 0.32 |
| ***Scrg1*** | 0.24 | 1.3 (1.1-1.5) | 8.5 | 0.0036 |
| *Slc6a15* | 0.0094 | 1 (0.86-1.2) | 0.01 | 0.91 |
| ***Pgf*** | 0.16 | 1.2 (1-1.4) | 3.7 | 0.053 |
| ***Bcar3*** | 0.28 | 1.3 (1.1-1.6) | 6.6 | 0.01 |
| *Gpx2* | 0.06 | 1.1 (0.93-1.2) | 0.83 | 0.36 |
| *Crispld2* | 0.057 | 1.1 (0.9-1.2) | 0.48 | 0.49 |
| *Csn2* | -0.023 | 0.98 (0.89-1.1) | 0.25 | 0.62 |
| *Btn1a1* | -0.008 | 0.99 (0.9-1.1) | 0.03 | 0.87 |
| ***Cd177*** | 0.12 | 1.1 (0.98-1.3) | 2.8 | 0.093 |
| *Cpe* | 0.061 | 1.1 (0.94-1.2) | 0.91 | 0.34 |
| *Csn1s1* | -0.05 | 0.95 (0.88-1) | 1.5 | 0.23 |
| ***Dpp10*** | 0.16 | 1.2 (1-1.4) | 4.6 | 0.031 |
| ***Unc79*** | 0.29 | 1.3 (1.1-1.7) | 7.5 | 0.0063 |
| *Cytip* | -0.17 | 0.84 (0.67-1.1) | 2.3 | 0.13 |
| ***Shc4*** | 0.47 | 1.6 (1.2-2) | 14 | 0.00019 |
| ***Slc35f1*** | 0.14 | 1.1 (0.94-1.4) | 1.9 | 0.17 |
| *Gbp3* | 0.084 | 1.1 (0.88-1.3) | 0.62 | 0.43 |
| *Muc15* | -0.061 | 0.94 (0.75-1.2) | 0.26 | 0.61 |
| *Csn1s2a* | -0.012 | 0.99 (0.9-1.1) | 0.07 | 0.79 |
| ***Mfsd4*** | 0.14 | 1.1 (0.94-1.4) | 1.9 | 0.17 |

**Table S6.** Some of the NCAPH signature genes obtained in mice were associated with the prognosis in patients (disease-free survival) with luminal A and luminal B tumors defined by PAM50. P values were obtained from the Kaplan-Meier curve using the Log-Rank test. The analyses were performed in the KM-plotter tool—red, significant P values.

|  | **Lum A (PAM50)** | | **Lum B (PAM50)** | |
| --- | --- | --- | --- | --- |
| Gene Symbol | KM (p) median | **Evolution & high levels** | KM (p) median | **Evolution & high levels** |
| *Ncaph* | 0.00049 (914/895) | Poor evolution | 0.99 (676/677) |  |
| *Oip5/CT86* | 0.23 (906/903) |  | 0.061 (678/675) |  |
| *Rad51/BRCC5* | 0.016 (931/878) | Poor evolution | 0.054 (693/660) |  |
| *Bub1b/SSK1* | 0.00024 (908/901) | Poor evolution | 0.0047 (677/676) | Poor evolution |
| *Casc5* | 0.17 (323/308) |  | 0.0085 (288/278) | Poor evolution |
| *Bub1* | 0.0018 (912/897) | Poor evolution | 0.14 (679/674) |  |
| *Cdc25c* | 0.11 (907/902) |  | 0.13 (679/674) |  |
| *Pole2* | 0.52 (908/901) |  | 0.76 (677/676) |  |
| *Rrm2* | 5.3e-05 (906/903) | Poor evolution | 0.022 (677/676) | Poor evolution |
| *Gjb1* | 0.36 (907/902) |  | 0.064 (677/676) |  |
| *Tspan1* | 0.049 (906/903) | Poor evolution | 0.68 (677/676) |  |
| *Psrc1* | 0.12 (904/905) |  | 0.38 (677/676) |  |
| *H2afy2* | 0.087 (906/903) |  | 0.31 (677/676) |  |
| *Krt23/CK23* | 0.18 (904/905) |  | 0.028 (678/675) | Good evolution |
| *Dpp10* | 0.74 (323/308) |  | 0.97 (285/281) |  |
| *Pde1c* | 0.26 (912/897) |  | 0.11 (679/674) |  |
| *Spink5/DKFZp686K19184* | 0.16 (909/900) |  | 0.076 (680/673) |  |
| *Higd1a* | 0.043 (316/315) | Good evolution | 0.00014 (284/282) | Good evolution |
| *Slc38a1* | 0.6 (316/315) |  | 0.57 (283/283) |  |
| *Wnt5a/hWNT5A* | 0.96 (907/902) |  | 0.1 (678/675) |  |
| *Angpt1/ANG1* | 0.053 (907/902) |  | 0.62 (679/674) |  |
| *Shc4* | 0.62 (320/311) |  | 0.41 (289/277) |  |
| *Mfsd4* | 0.31 (318/313) |  | 0.08 (285/281) |  |
| *Sftpd* | 0.058 (921/888) |  | 0.34 (701/652) |  |
| *Unc79* | 0.042 (320/311) | Good evolution | 0.97 (286/280) |  |
| *Prom1* | 0.41 (906/903) |  | 0.28 (676/677) |  |
| *Slc38a3* | 0.89 (910/899) |  | 0.41 (681/672) |  |
| *Cytip/CYBR* | 0.17 (907/902) |  | 0.055 (678/675) |  |
| *Pik3c2g* | 0.65 (904/905) |  | 0.15 (679/674) |  |
| *Spp1/HsT2645* | 0.017 (904/905) | Poor evolution | 0.099 (676/677) |  |
| *Slc35f1* | 0.63 (320/311) |  | 0.35 (284/282) |  |
| *Serpinb5* | 0.73 (905/904) |  | 0.51 (680/673) |  |
| *Pglyrp1/TNFSF3L* | 0.38 (991/818) |  | 0.7 (736/617) |  |
| *Scrg1* | 0.15 (908/901) |  | 0.11 (676/677) |  |
| *Slc6a15* | 0.67 (321/310) |  | 0.00027 (292/274) | Good evolution |
| *Pgf* | 0.45 (908/901) |  | 0.16 (677/676) |  |
| *Bcar3* | 0.51 (904/905) |  | 0.23 (676/677) |  |
| *Gbp3* | 0.13 (316/315) |  | 0.47 (283/283) |  |
| *Gpx2* | 0.69 (905/904) |  | 0.24 (677/676) |  |
| *Crispld2* | 0.94 (904/905) |  | 0.82 (676/677) |  |
| *Muc15* | 0.11 (317/314) |  | 0.44 (288/278) |  |
| *Csn2* | 0.35 (909/900) |  | 0.12 (682/671) |  |
| *Csn1s2a/CSN1S2AP* | 0.98 (316/315) |  | 0.001 (292/274) | Good evolution |
| *Btn1a1* | 0.085 (910/899) |  | 0.061 (679/674) |  |
| *Cd177* | 0.02 (904/905) | Good evolution | 0.14 (677/676) |  |
| *Cpe* | 0.99 (905/904) |  | 0.058 (676/677) |  |
| *Csn1s1* | 0.16 (913/896) |  | 0.3 (681/672) |  |
| *Rdh12* | 0.74 (322/309) |  | 0.9 (289/277) |  |

**Table S7.** Bivariant analyses using Cox regression to identify associations between the transcript levels associated with NCAPH and relapse-free survival (RFS) in the human GOBO cohort with luminal A breast cancer. Transcripts with a P-value lower than 0.25 were chosen to construct the multivariate model using LASSO regression.

|  | **Cox regression** | | | |
| --- | --- | --- | --- | --- |
|  | **Beta** | **HR (95% CI for HR)** | **Wald test** | **p.value** |
| ***NCAPH*** | 0.17 | 1.2 (0.97-1.5) | 2.7 | 0.1 |
| *OIP5* | -0.018 | 0.98 (0.74-1.3) | 0.02 | 0.9 |
| *RAD51* | -0.041 | 0.96 (0.81-1.1) | 0.24 | 0.63 |
| ***BUB1B*** | 0.48 | 1.6 (1-2.5) | 4.5 | 0.034 |
| *CASC5* | -0.085 | 0.92 (0.78-1.1) | 1.1 | 0.3 |
| ***BUB1*** | 0.3 | 1.4 (0.96-1.9) | 3 | 0.082 |
| *CDC25C* | 0.072 | 1.1 (0.88-1.3) | 0.49 | 0.48 |
| *POLE2* | -0.13 | 0.88 (0.54-1.4) | 0.27 | 0.61 |
| *GJB1* | 0.018 | 1 (0.76-1.4) | 0.02 | 0.9 |
| *PSRC1* | 0.31 | 1.4 (0.79-2.4) | 1.2 | 0.27 |
| ***RRM2*** | 0.31 | 1.4 (1.1-1.8) | 5.5 | 0.019 |
| ***TSPAN1*** | 0.13 | 1.1 (0.96-1.3) | 2.3 | 0.13 |
| ***H2AFY2*** | -0.17 | 0.84 (0.71-1) | 3.7 | 0.053 |
| ***KRT23*** | -0.091 | 0.91 (0.79-1.1) | 1.5 | 0.22 |
| ***HIGD1A*** | -0.46 | 0.63 (0.47-0.86) | 8.8 | 0.0031 |
| *SPINK5* | -0.07 | 0.93 (0.74-1.2) | 0.35 | 0.56 |
| *ANGPT1* | 0.044 | 1 (0.74-1.5) | 0.06 | 0.8 |
| ***SLC38A1*** | -0.22 | 0.8 (0.65-0.99) | 4.1 | 0.044 |
| *WNT5A* | 0.061 | 1.1 (0.84-1.3) | 0.25 | 0.61 |
| *PROM1* | 0.081 | 1.1 (0.92-1.3) | 0.94 | 0.33 |
| *PGLYRP1* | 0.061 | 1.1 (0.78-1.4) | 0.15 | 0.7 |
| ***PIK3C2G*** | -0.17 | 0.84 (0.68-1) | 2.8 | 0.096 |
| ***SLC38A3*** | 0.18 | 1.2 (0.93-1.5) | 2 | 0.16 |
| *SPP1* | -0.046 | 0.96 (0.78-1.2) | 0.2 | 0.66 |
| *CD177* | -0.11 | 0.89 (0.73-1.1) | 1.2 | 0.28 |
| *BCAR3* | -0.12 | 0.88 (0.66-1.2) | 0.65 | 0.42 |
| *SERPINB5* | 7.50E-05 | 1 (0.88-1.1) | 0 | 1 |
| *SLC6A15* | 0.11 | 1.1 (0.87-1.4) | 0.79 | 0.37 |
| *SCRG1* | -0.0079 | 0.99 (0.68-1.5) | 0 | 0.97 |
| ***GPX2*** | 0.24 | 1.3 (0.99-1.6) | 3.7 | 0.055 |
| *PGF* | 0.033 | 1 (0.8-1.3) | 0.06 | 0.8 |
| *CRISPLD2* | -0.045 | 0.96 (0.73-1.3) | 0.11 | 0.74 |
| *CSN2* | -0.13 | 0.88 (0.69-1.1) | 1.1 | 0.28 |
| *BTN1A1* | -0.037 | 0.96 (0.81-1.1) | 0.17 | 0.68 |
| ***CPE*** | 0.23 | 1.3 (0.94-1.7) | 2.4 | 0.12 |
| *CSN1S1* | -0.079 | 0.92 (0.79-1.1) | 0.89 | 0.35 |
| ***SFTPD*** | -0.11 | 0.9 (0.75-1.1) | 1.5 | 0.22 |

**Table S8.** List of genes whose transcripts are components of the Gene Signature for Luminal A 10 (GSLA10).

| **Gene** | **Description** | **References** |
| --- | --- | --- |
| ***NCAPH*** | Non-structural maintenance of Chromosome (SMC) condensin I complex subunit H: This gene is a component of the condensin complex, which is responsible for the condensation and stabilization of chromosomes during mitosis and meiosis. Mutations can be associated with cancers. |  |
| ***BUB1*** | BUB1 Mitotic Checkpoint Serine/Threonine Kinase: This gene plays an important role in the spindle checkpoint function during the cell cycle, preventing cells with damaged DNA from undergoing mitosis. Mutations are linked to various types of cancer. | [1,2] |
| ***RRM2*** | Ribonucleotide reductase subunit M2: This gene plays a critical role in DNA replication and repair, as it  is responsible for the biosynthesis of deoxyribonucleotides from ribonucleotides. Its overexpression has been associated with cancer proliferation. | [3,4] |
| ***KRT23*** | Keratin 23: Keratins are a family of intermediate filament proteins responsible for maintaining the structural integrity of epithelial cells. Krt23 is often involved in processes like cell differentiation and disease, including cancer. | [5] |
| ***HIGD1A*** | HIG1 domain family member 1A: It's a protein-coding gene. Diseases associated with HIGD1A include Mitochondrial Complex IV deficiency and Leigh Syndrome. | [6] |
| ***SLC38A3*** | Solute carrier family 38 member 3; This gene is part of a family of transport proteins that carry specific molecules across cell membranes. It is specifically known to function as a sodium-coupled neutral amino acid transporter. | [7] |
| ***PIK3C2G*** | Phosphatidylinositol 3-kinase catalytic class 2 (γ) subunit: It's an enzyme that in humans is  encoded by the PIK3C2G gene. It has been linked with roles in cell growth, proliferation, differentiation, motility, survival, and intracellular trafficking. | [8] |
| ***SFTPD*** | Surfactant protein D: This gene provides instructions for making a protein found in the lungs that plays a critical role in lung function and the innate immune response. | [9] |
| ***CPE*** | Carboxypeptidase E is involved in the biosynthesis of peptide hormones and neurotransmitters. This protein may also function independently of its peptidase activity, as a neurotrophic factor that promotes neuronal survival, and as a sorting receptor that binds to regulated secretory pathway proteins. | [10-13] |
| ***GPX2*** | Glutathione peroxidase 2; This gene encodes an enzyme that is part of the body's antioxidant defense system, protecting cells from damage by free radicals. | [14] |

# References

- 1. Wang, Z., Katsaros, D., Shen, Y., Fu, Y., Canuto, E. M., Benedetto, C., Lu, L., Chu, W. M., Risch, H. A., & Yu, H. (2015). Biological and clinical significance of MAD2L1 and BUB1, genes frequently appearing in expression signatures for breast cancer prognosis. PLoS ONE, 10(8). https://doi.org/10.1371/journal.pone.0136246.
  2. Han, J. Y., Han, Y. K., Park, G. Y., Kim, S. D., Kim, J. S., Jo, W. S., & Geun Lee, C. (2015). Bub1 is required for maintaining cancer stem cells in breast cancer cell lines. Scientific Reports, 5. https://doi.org/10.1038/srep15993.
  3. Abdel-Rahman, M. A., Mahfouz, M., & Habashy, H. O. (2022). RRM2 expression in different molecular subtypes of breast cancer and its prognostic significance. Diagnostic Pathology, 17(1). https://doi.org/10.1186/s13000-021-01174-4.
  4. Shi, S. C., Zhang, Y., & Wang, T. (2022). High RRM2 expression has poor prognosis in specific types of breast cancer. PLoS ONE, 17(3 March). https://doi.org/10.1371/ journal.pone.0265195.
  5. Zhou, J., Qian, W., Huang, C., Mai, C., Lai, Y., Lin, Z., & Lai, G. (2022). Combined targeting of KRT23 and NCCRP1 as a potential novel therapeutic approach for the treatment of triple-negative breast cancer. Gland Surgery, 11(10), 1673–1682. https://doi.org/10.21037/ gs-22-486.

9

**References Table S8 (cont.)**

- 1. Ameri, K., Rajah, A. M., Nguyen, V., Sanders, T. A., Jahangiri, A., DeLay, M., Donne, M., Choi,

H. J., Tormos, K. v., Yeghiazarians, Y., Jeffrey, S. S., Rinaudo, P. F., Rowitch, D. H., Aghi, M., & Maltepe, E. (2013). Nuclear Localization of the Mitochondrial Factor HIGD1A during Metabolic Stress. PLoS ONE, 8(4). https://doi.org/10.1371/journal.pone.0062758.

- 1. Zhao, X., Jin, L., Liu, Y., Liu, Z., & Liu, Q. (2022). Bioinformatic analysis of the role of solute carrier-glutamine transporters in breast cancer. Annals of Translational Medicine, 10(14), 777–777. https://doi.org/10.21037/atm-22-2620.
  2. Chu, C. A., Wang, Y. W., Chen, Y. L., Chen, H. W., Chuang, J. J., Chang, H. Y., Ho, C. L., Chang, C., Chow, N. H., & Lee, C. T. (2021). The role of phosphatidylinositol 3-kinase catalytic subunit type 3 in the pathogenesis of human cancer. International Journal of Molecular Sciences, 22(20), 10964. https://doi.org/10.3390/ijms222010964.
  3. Umeda, Y., Hasegawa, Y., Otsuka, M., Ariki, S., Takamiya, R., Saito, A., Uehara, Y., Saijo, H., Kuronuma, K., Chiba, H., Ohnishi, H., Sakuma, Y., Takahashi, H., Kuroki, Y., & Takahashi, M. (2017). Surfactant protein D inhibits activation of non-small cell lung cancer-associated mutant EGFR and affects clinical outcomes of patients. Oncogene, 36(46), 6432–6445. https:// doi.org/10.1038/onc.2017.253.
  4. Fan, S., Li, X., Li, L., Wang, L., Du, Z., Yang, Y., Zhao, J., & Li, Y. (2016). Silencing of carboxypeptidase E inhibits cell proliferation, tumorigenicity, and metastasis of osteosarcoma cells. OncoTargets and Therapy, 9, 2795–2803. https://doi.org/10.2147/OTT.S98991.
  5. Skalka, N., Caspi, M., Caspi, E., Loh, Y. P., & Rosin-Arbesfeld, R. (2013). Carboxypeptidase E: A negative regulator of the canonical Wnt signaling pathway. Oncogene, 32(23), 2836–2847. https:// doi.org/10.1038/onc.2012.308.
  6. Cheng, Y., Cawley, N. X., & Loh, Y. P. (2013). Carboxypeptidase E/NFα1: A New Neurotrophic Factor against Oxidative Stress-Induced Apoptotic Cell Death Mediated by ERK and PI3-K/AKT Pathways. PLoS ONE, 8(8). https://doi.org/10.1371/journal.pone.0071578.
  7. Hareendran, S., Albraidy, B., Yang, X., Liu, A., Breggia, A., & Loh, Y. P. (2021). Exosomal Carboxypeptidase E (CPE) and CPE-shRNA Loaded Exosomes Control Growth and Invasion of Recipient Hepatocellular Carcinoma Cells. International of Molecular Sciences, 23 (6), 3113. https://doi.org/10.21203/rs.3.rs-335388/v1.
  8. Naiki-Ito, A., Asamoto, M., Hokaiwado, N., Takahashi, S., Yamashita, H., Tsuda, H., Ogawa, K., & Shirai, T. (2007). Gpx2 is an overexpressed gene in rat breast cancers induced by three different chemical carcinogens. Cancer Research, 67(23), 11353–11358. https://

doi.org/10.1158/0008-5472.CAN-07-2226.
