# Supplementary material for: NCAPH drives breast cancer progression and identifies a gene signature that predicts luminal a tumour recurrence
Source: Clin Transl Med. 2024 Feb 12;14(2):e1554. doi: 10.1002/ctm2.1554 (PMC10859882; doi:10.1002/ctm2.1554)
Supplement: Supplementary file 3 — Additional File 3 — Supplementary Figures. FIGURE S1. NCAPH and breast cancer prognosis: a preliminary analysis. FIGURE S2. Effect of NCAPH induction on breast cancer cells of basal origin. FIGURE S3. Comparative analysis of pERBB2 and ERBB2 levels in MMTV‐ErbB2 and MMTV‐NcaphErbB2 mouse tumours and quantification of Caspase 3. FIGURE S4. Analysis of Ncaph levels in local tumour growth response and metastatic potential post‐chemotherapy. FIGURE S5. The gene signature is correlated with intratumoural Ncaph levels in the BX‐Neu+ mouse cohort. FIGURE S6. The least absolute shrinkage and selection operator (LASSO) regression model is used to predict prognosis in luminal A breast cancer. [file CTM2-14-e1554-s001.docx]

**Supplemental Figure Legends**

**
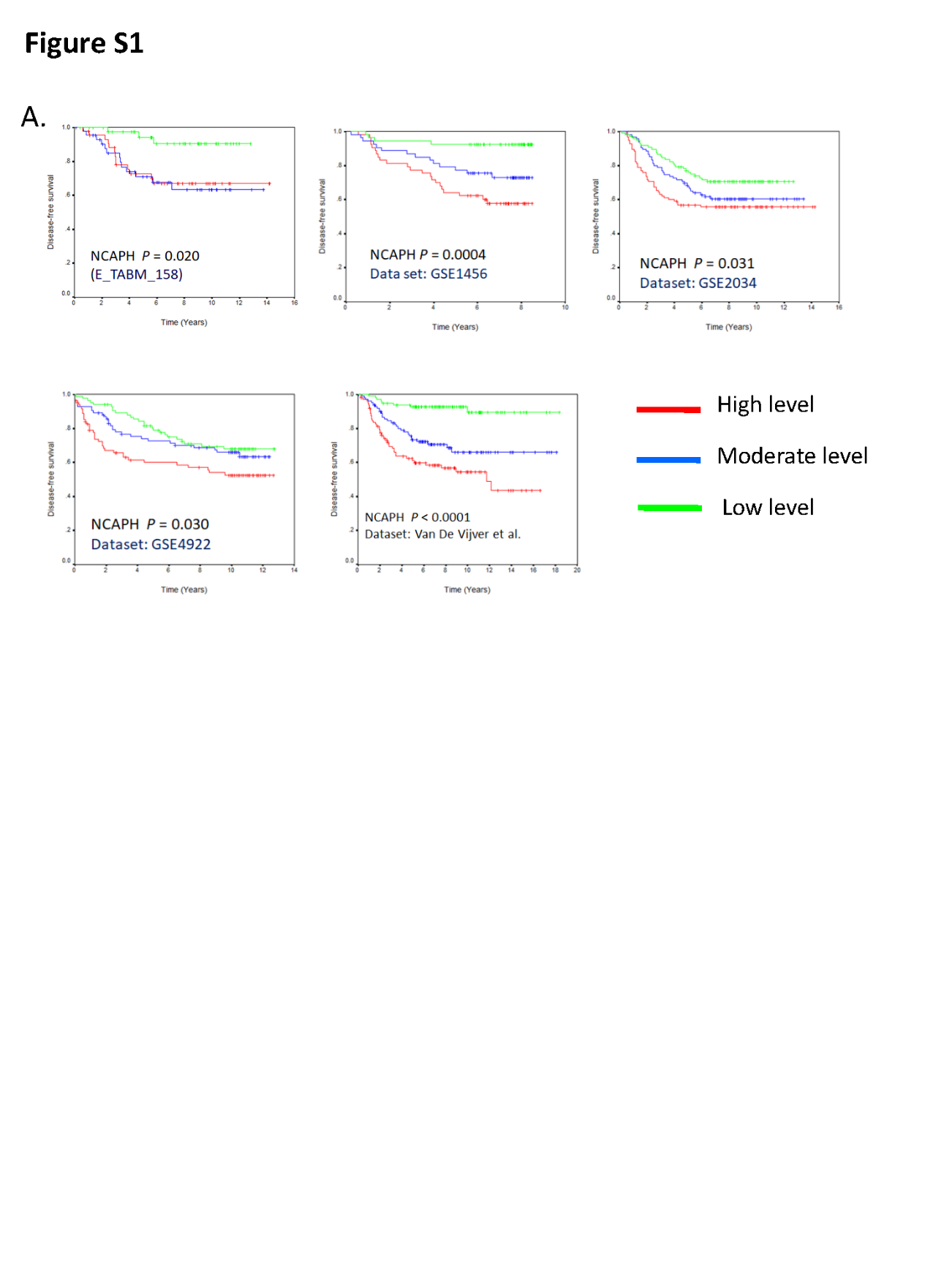
**

**Figure S1. NCAPH and Breast Cancer Prognosis: A Preliminary Analysis.** Associations between intratumoral NCAPH levels and breast cancer progression were examined across multiple databases, using Kaplan-Meier and Log-Rank tests for prognosis assessment. Within these gene expression datasets, genes differentially expressed during cell mitosis and linked to poor breast cancer prognosis were identified. Intratumoral NCAPH expression levels were categorized as low, intermediate, or high based on tertiles. Each graph denotes the originating dataset, and corresponding references are provided in the main text. The patient enrollment numbers for each study are as follows: E_TABM_158 with 176 participants (N=176), GSE1456 with 159 participants (N=159), GSE2034 with 286 participants (N=286), GSE4922 with 347 participants (N=347), and the study by van de Vijver et al. (2002) with 295 participants (N=295).


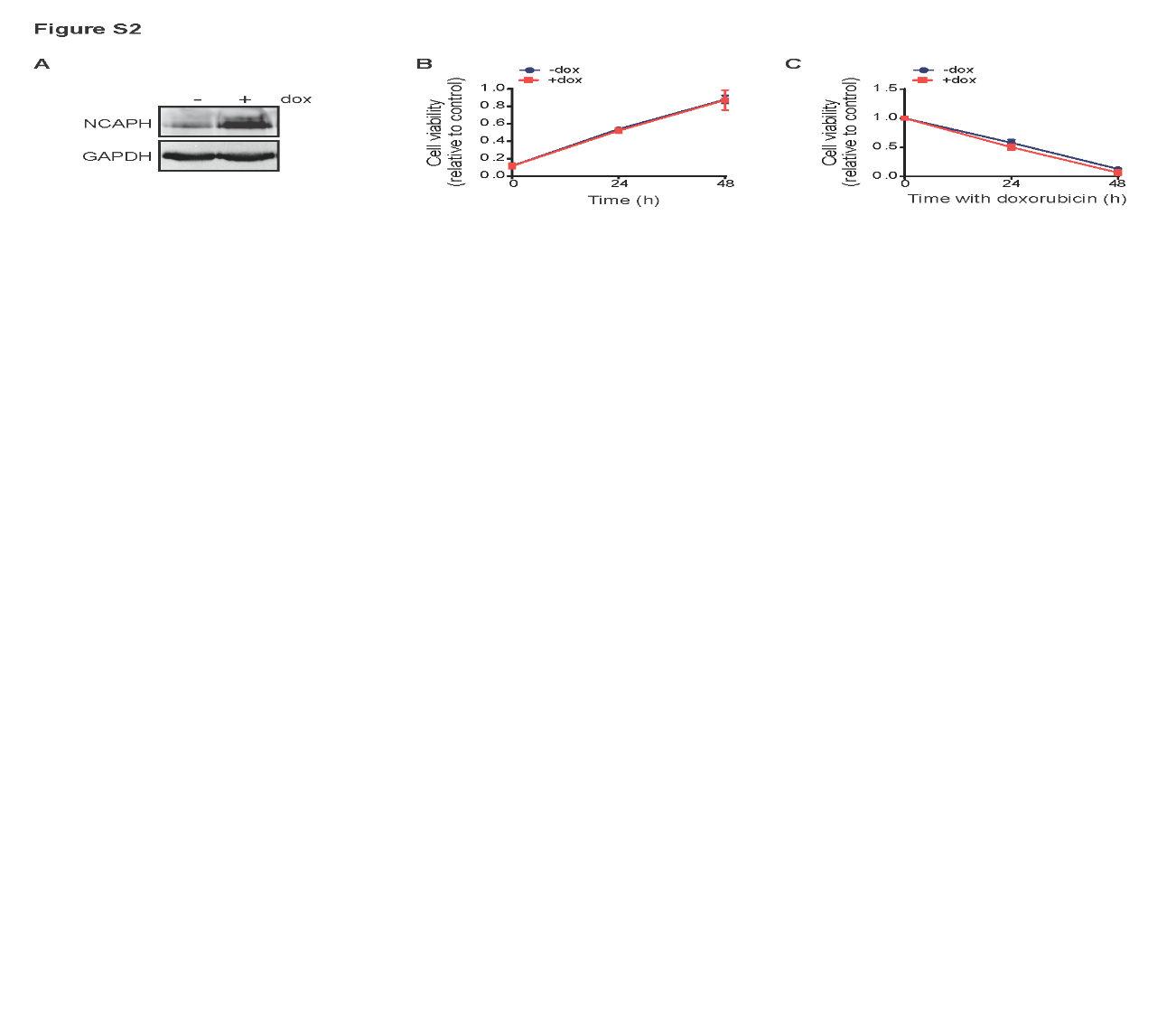


**Figure S2. Effect of NCAPH induction on breast cancer cells of basal origin.** **A**) Induction of NCAPH expression in the BT549 basal cell line. **B, C**) Evaluation of cell viability by MTT after NCAPH induction of the *TET-ON* promoter by doxycycline (+dox): at basal conditions, 24 and 48 hours (b) and after doxorubicin chemotherapy (c).


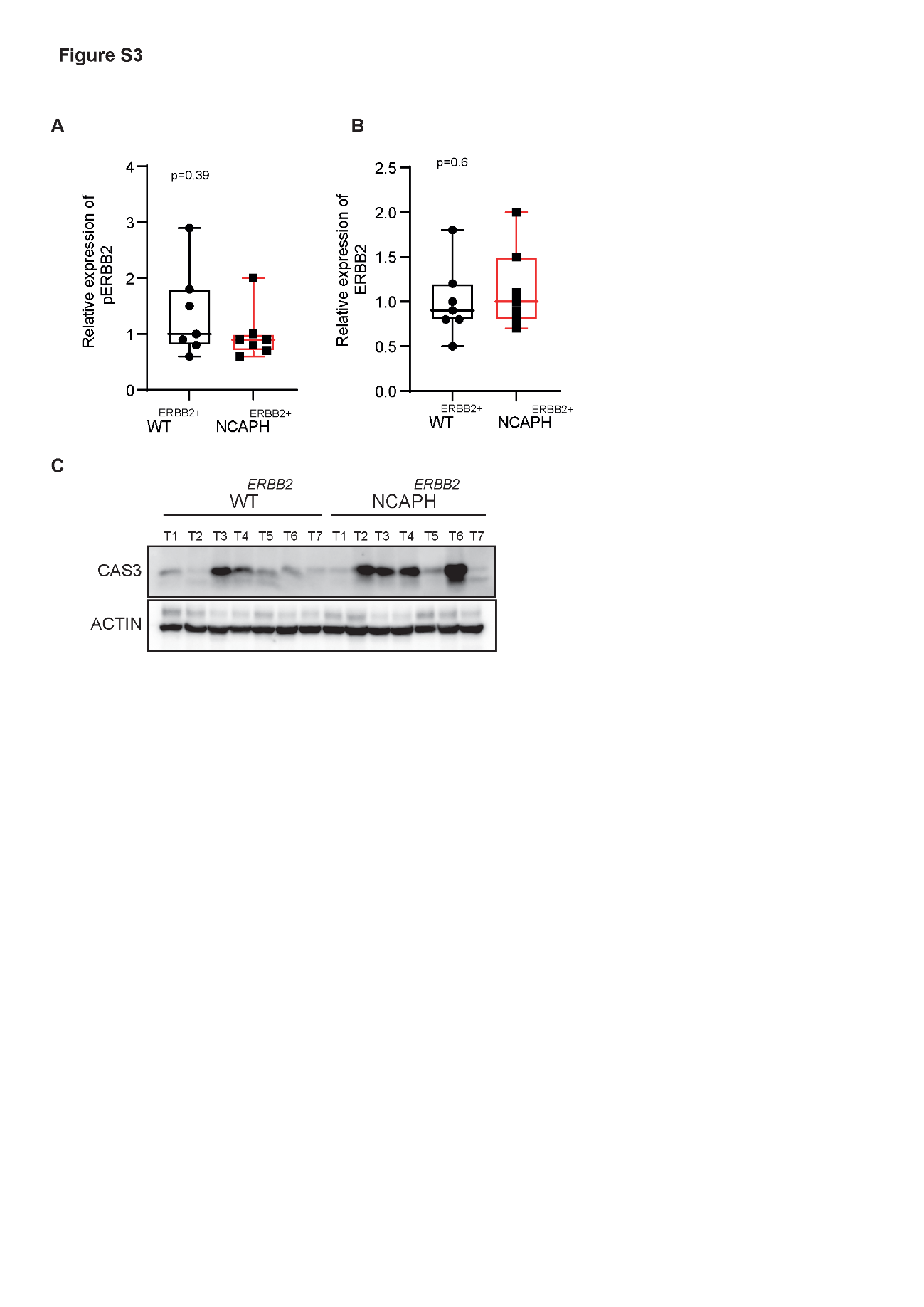


**Figure S3: Comparative analysis of ERBB2 and pERBB2 levels in MMTV-*ErbB2* and MMTV-*Ncaph^ErbB2^* mouse tumors, and Quantification of cleaved Caspase 3.** **A, B**) This figure presents the quantification of phospo-ERBB2 (pERBB2) (A) and total ERBB2 (B) levels, as determined by Western blotting and densitometry. The analysis shows no significant differences in ERBB2 or pERBB2 levels between tumors from MMTV-ErbB2 mice and those from double transgenic MMTV-*Ncaph^ErbB2^* mice. **C**) Panel C depicts the quantification of cleaved Caspase 3 levels in tumors from the same groups of mice.


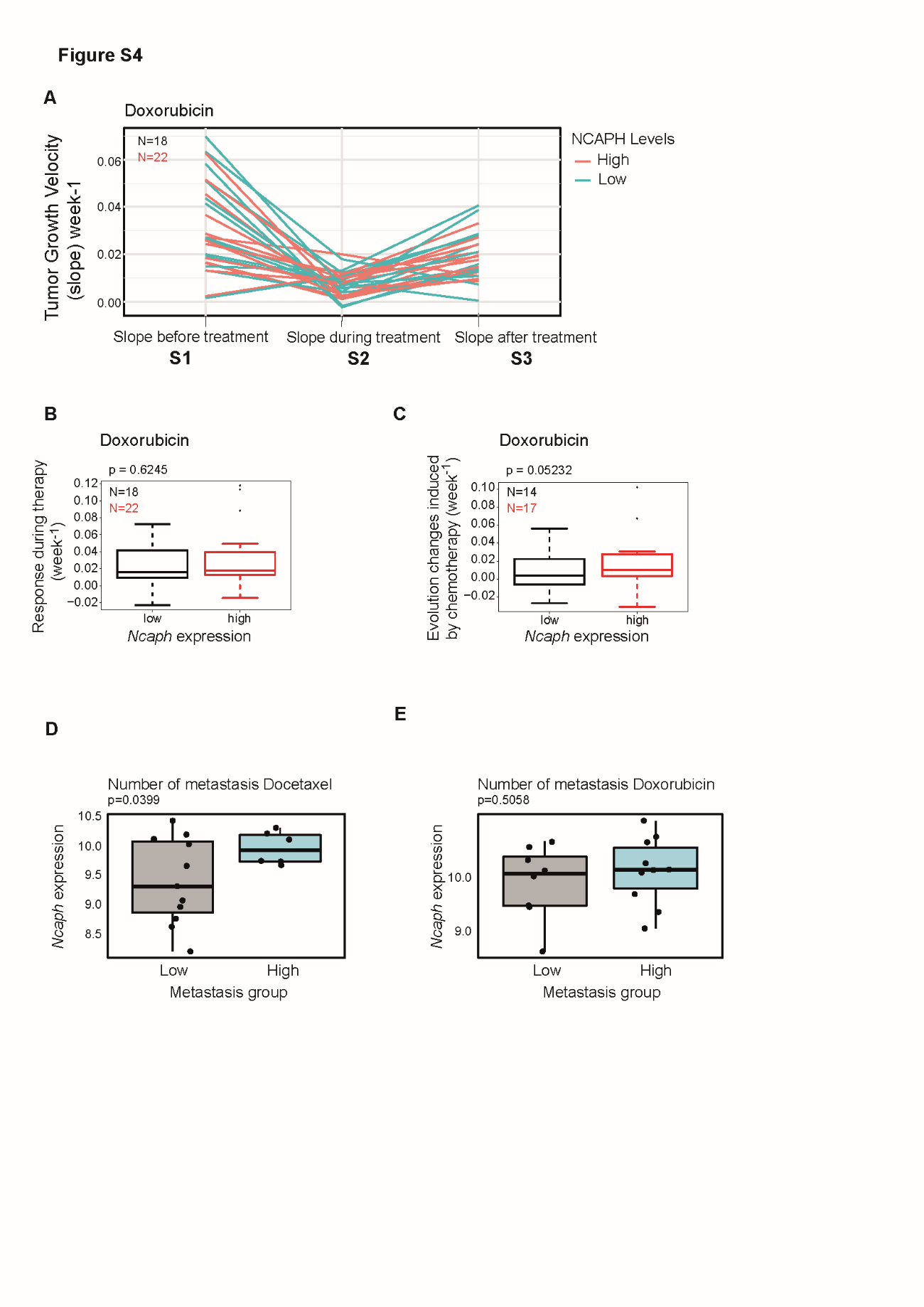


**Figure S4. Analysis of *Ncaph* levels in local tumor growth response and metastatic potential post-chemotherapy.** **A-C**) Impact of doxorubicin on local tumor growth based on *Ncaph* levels. **A**) Illustrates the growth curve's slope at two critical points during doxorubicin treatment. Point S1 represents the slope before chemotherapy, and Point S2 indicates the slope during chemotherapy, typically corresponding to the tumor's maximum response (lowest slope). The Response During Treatment (RDT) is calculated as the difference between slopes S1 and S2, with a higher RDT value indicating a better treatment response. **B**) This panel compares RDT between tumors with high and low *Ncaph* levels (based on external tertiles). **C**) This panel examines changes in tumor behavior in terms of local growth post-chemotherapy cessation (Evolution Changes Induced by Chemotherapy, ECIC) by comparing slopes S1 and S3 (S3 is the slope after chemotherapy). **D)** This panel analyzes the difference in *Ncaph* levels in tumors post-docetaxel treatment, categorizing them based on their metastatic potential (0 or 2 metastasis indicating a better response, and 3 or more metastases indicating a poorer response). Tumors with high *Ncaph* levels showed more resistance to docetaxel in preventing metastasis, while those with low *Ncaph* levels exhibited fewer metastases post-treatment. **E)** This panel focuses on the doxorubicin treatment group, revealing no significant differences in *Ncaph* levels between the two categories of metastatic potential. However, a more detailed Poisson GLM regression analysis, considering the entire sample, does reveal a positive effect of doxorubicin in reducing tumor spread (refer to main **Figure 4J**). In panels B-E, low and high *Ncaph* levels were defined according to the first and third tertiles, respectively.

**
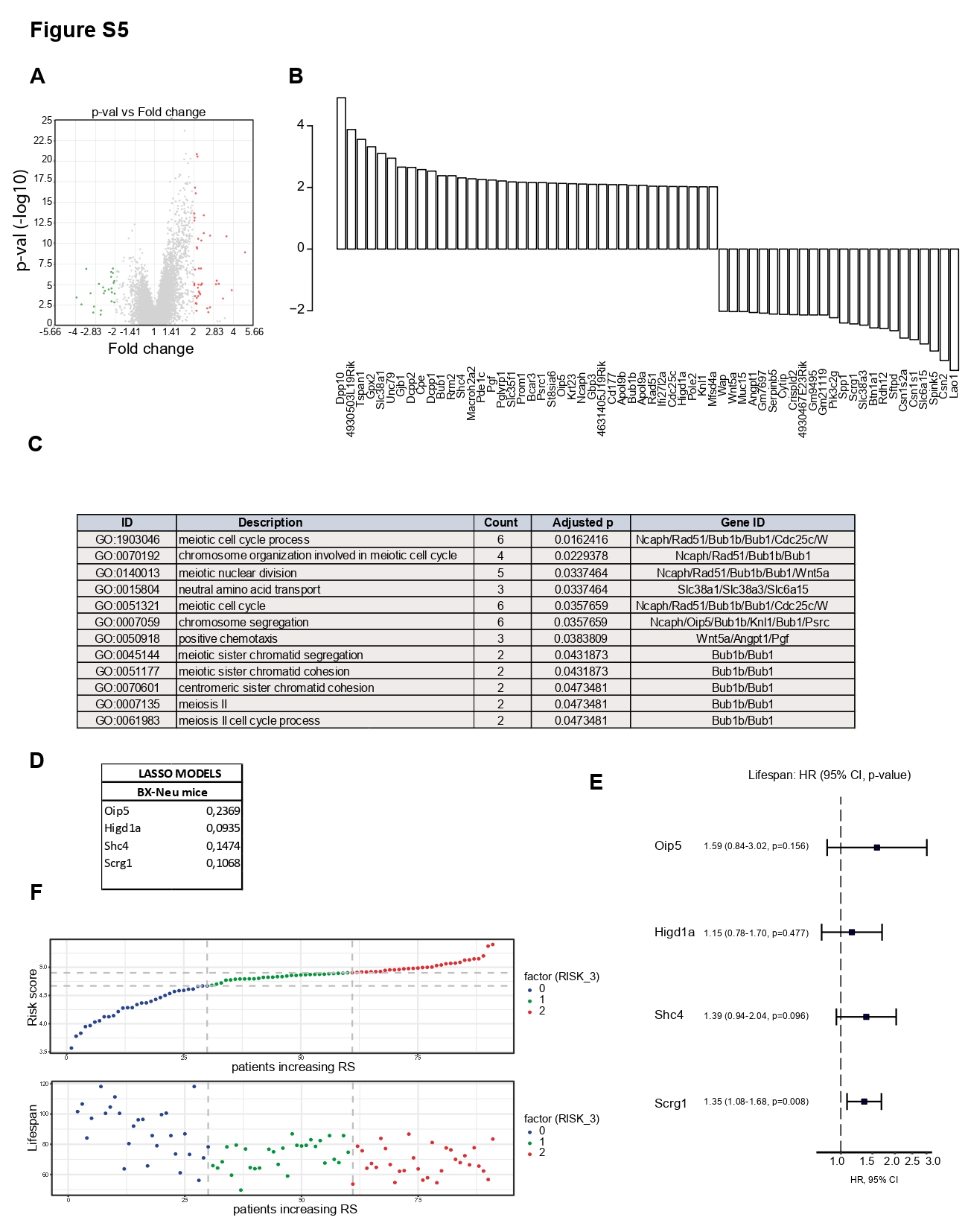
**

**Figure S5.** **Gene signature is correlated with intratumoral Ncaph levels in the BX-*Neu+* mouse cohort. A)** A volcano plot identifies the 64-gene signature associated with intratumor levels of *Ncaph.* **B)** Upregulated and downregulated genes were associated with high levels of intratumoral *Ncaph*. **C)** Summary of the enrichment analysis of the signature genes associated with intratumoral levels of *Ncaph*. Several of the genes are associated with mitosis. **D)** Genes selected for the human risk model **E)** Definition of the risk of poor survival according to tertiles in BX-*Neu+* mice. **F)** Individual contribution to the risk of each gene implicated in the LASSO model: HR, hazard ratio. Risk levels - low, medium, and high - were categorized using tertile divisions, as detailed in the panel.


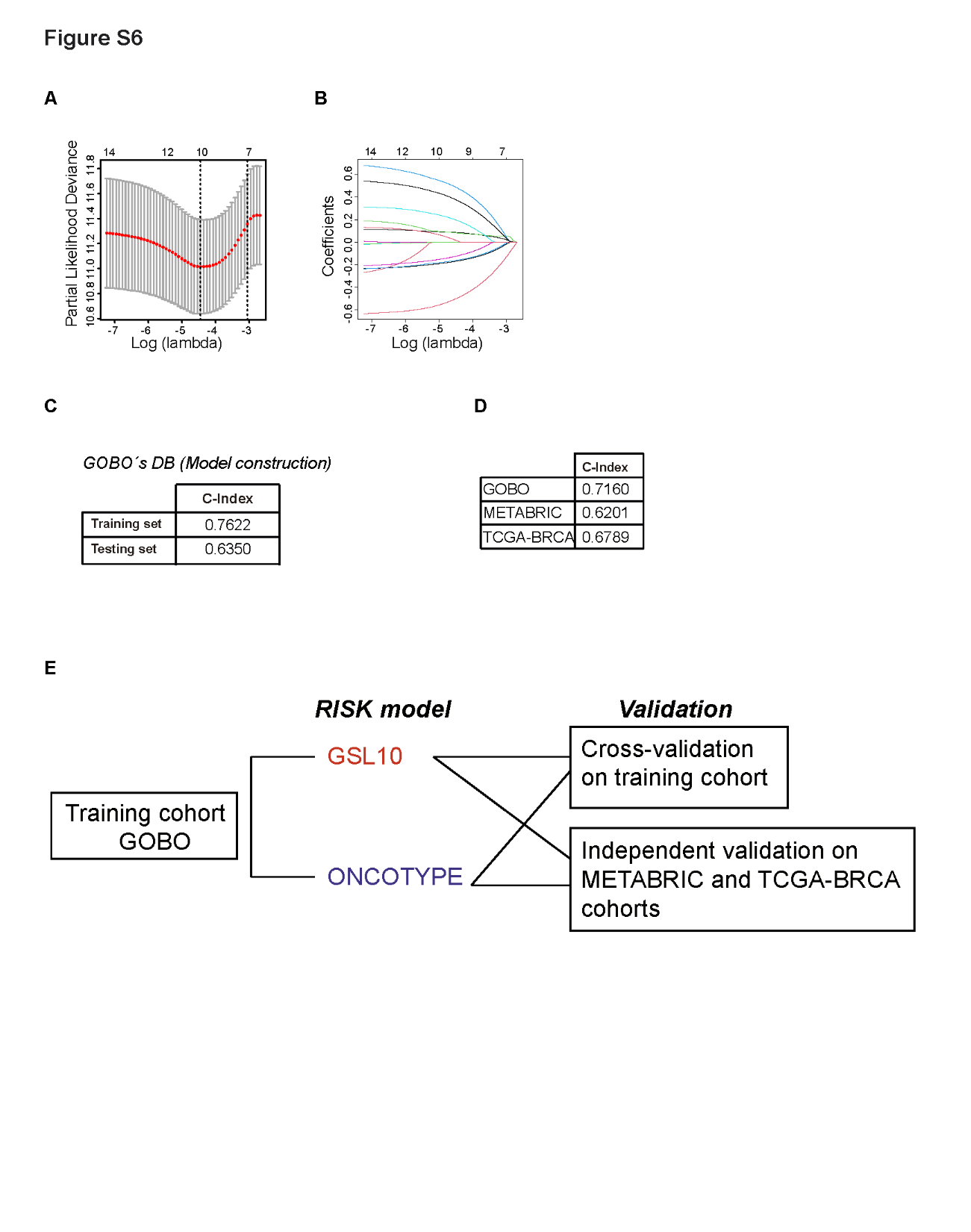


**Supplementary Figure S6. The least absolute shrinkage and selection operator (LASSO) regression model is used to predict prognosis in luminal A breast cancer.** Generation of a LASSO multivariate regression model for the definition of prognosis in luminal A tumors. The model was generated in a database of luminal A tumors defined by PAM50 (GOBO), where the LASSO GSLA10 signature was identified. **A)** Regression coefficient map of genes in the LASSO model. Cross-validation was used to select the optimal tuning parameter (λ), and the dotted vertical lines were drawn at the optimal values using the minimum criteria and the 1- standard error (SE) of the minimum criteria (1-SE criteria)**.** **B)** The graph shows the screening path of the LASSO regression model. Each curve represents a LASSO coefficient of the 45 prognostic genes, and the x-axis indicates the regularization penalty parameter. When the number of variables was 10, the partial likelihood deviation was at the minimum, corresponding to the minimum λ value**.** **C)** Goodness-of-fit measures for the generated LASSO models in the human database (GOBO). Training and testing model results from the GOBO database. C-index is the concordance correlation coefficient. **D)** Global results for GOBO, METABRIC and TCGA-BRCA databases. C-index is the concordance correlation coefficient. **E)** The study design for evaluation, validation, and comparison of GSLA10 and Oncotype signatures on GOBO, METABRIC, and TCGA-BRCA cohorts for the definition of prognosis in luminal A tumors.
